# Vertically grown ultrathin Bi_2_SiO_5_ as high-*κ* single-crystalline gate dielectric

**DOI:** 10.1038/s41467-023-40123-1

**Published:** 2023-07-21

**Authors:** Jiabiao Chen, Zhaochao Liu, Xinyue Dong, Zhansheng Gao, Yuxuan Lin, Yuyu He, Yingnan Duan, Tonghuai Cheng, Zhengyang Zhou, Huixia Fu, Feng Luo, Jinxiong Wu

**Affiliations:** 1 Tianjin Key Lab for Rare Earth Materials and Applications, Center for Rare Earth and Inorganic Functional Materials, Smart Sensor Interdisciplinary Science Center, School of Materials Science and Engineering, Nankai University, Tianjin, 300350 China; 2 State Key Laboratory of High Performance Ceramics and Superfine Microstructure, Shanghai Institute of Ceramics, Chinese Academy of Sciences, Shanghai, 200093 China; 3 Center of Quantum Materials and Devices & College of Physics, Chongqing University, Chongqing, 401331 China

**Keywords:** Electronic devices, Two-dimensional materials

## Abstract

Single-crystalline high-*κ* dielectric materials are desired for the development of future two-dimensional (2D) electronic devices. However, curent 2D gate insulators still face challenges, such as insufficient dielectric constant and difficult to obtain free-standing and transferrable ultrathin films. Here, we demonstrate that ultrathin Bi_2_SiO_5_ crystals grown by chemical vapor deposition (CVD) can serve as excellent gate dielectric layers for 2D semiconductors, showing a high dielectric constant (>30) and large band gap (~3.8 eV). Unlike other 2D insulators synthesized via in-plane CVD on substrates, vertically grown Bi_2_SiO_5_ can be easily transferred onto other substrates by polymer-free mechanical pressing, which greatly facilitates its ideal van der Waals integration with few-layer MoS_2_ as high-*κ* dielectrics and screening layers. The Bi_2_SiO_5_ gated MoS_2_ field-effect transistors exhibit an ignorable hysteresis (~3 mV) and low drain induced barrier lowering (~5 mV/V). Our work suggests vertically grown Bi_2_SiO_5_ nanoflakes as promising candidates to improve the performance of 2D electronic devices.

## Abstract (web summary)

Crystalline high-κ dielectric materials are desired for the development of future 2D electronic devices. Here, the authors report the in-plane and out-of-plane chemical vapor deposition growth of ultrathin Bi_2_SiO_5_ crystals with dielectric constant >30 and a band gap of ~3.8 eV, showing their effective application as gate dielectric layers of MoS_2_ transistors.

## Introduction

Two-dimensional (2D) semiconductors hold great promise for fabricating more-than-Moore transistors and exploring the emergent transport properties^1–5^, but achieving their theoretical performance also requires compatible high-*k* dielectrics to guarantee efficient gate control^6–10^. Compared to traditional amorphous dielectrics (Al_2_O_3_ and HfO_2_)^11–13^, single-crystalline gate insulators with dangling-bond-free surfaces are more competitive to fabricate high-performance 2D devices with reduced interfacial scatterings and gate hysteresis^14–18^. For example, hexagonal boron nitride (*h*-BN) has been widely used as a van der Waals (vdWs) substrate to improve carrier mobility and investigate exotic properties of 2D materials^19–23^. However, *h*-BN is also well known for its shortcomings of low dielectric constant (*k* = 2~4) and harsh conditions (pressure: >4 G Pa, temperature: >1673 K) for the growth of high-quality single crystals^24^. Therefore, it is highly desirable to discover new vdWs insulators similar to *h*-BN, but has a much higher dielectric constant and more facile synthetic conditions, for exploring the emergent transport properties of 2D materials in a high-*κ* dielectric environment, as well as for fabricating 2D-material-based electronic devices with scaled supply voltage^15,16^. Even so, very rare have succeeded^18,25^. Very recently, Peng et al.^18^ reported the synthesis of Bi_2_SeO_5_ bulk crystals grown by chemical vapor transport (CVT), which can be exfoliated into few-nanometer-thick nanosheets and serve as an “*h*-BN” like high-*k* dielectric to improve the mobility of 2D materials and enable the observation of quantum Hall effects in Bi_2_O_2_Se. Nevertheless, the CVT process for the synthesis of Bi_2_SeO_5_ bulk crystals is also very time-consuming (typically ~40 days). Besides, similar to other vdWs dielectrics, transferable Bi_2_SeO_5_ nanoflakes with suitable thicknesses and domain sizes were also prepared by a low-efficiency method of mechanical exfoliation for subsequent vdWs device integration.

Compared to mechanical exfoliation, direct growth of free-standing ultrathin 2D insulators with high-*k* nature by chemical vapor deposition (CVD) is much more efficient, but remains challenging. Typically, CVD-grown atomically thin 2D insulators with a layered crystal structure preferably adopt an in-plane growth mode on substrates^26–29^, which will inevitably set obstacles for clean sample transfer and subsequent vdWs integration. However, if an ultrathin 2D insulator can be vertically grown on the substrate, just like the case in Bi_2_O_2_Se^3,30,31^, the transfer problem can be easily overcome due to much reduced interfacial interaction.

Bi_2_SiO_5_ is a well-known high-*κ* dielectrics with an Aurivillius-type layered structure and a large band gap of 3.5~4.4 eV^32–35^. According to the previous works regarding the bulk single crystals and polycrystalline powders of Bi_2_SiO_5_, Bi_2_SiO_5_ shows an anisotropic dielectric constant^36,37^ and its out-of-plane dielectric constant can be as high as 30~80^36–39^, and thus was suggested as a potential candidate for high-temperature dielectrics^39^. Here, Bi_2_SiO_5_ was demonstrated as an excellent gate dielectric for 2D semiconductors. Ultrathin Bi_2_SiO_5_ single crystals with thickness down to monolayer were successfully synthesized by a facile CVD method, concurrently owing to the high dielectric constant (>30), large band gap (~3.8 eV), and large breakdown field strength. Remarkably, the preferable CVD growth mode of Bi_2_SiO_5_ can be regulated from in-plane to out-of-plane under optimized conditions, showing great feasibility on sample transfer by polymer-free mechanical pressing. Using ultrathin Bi_2_SiO_5_ nanoflakes as the vdW dielectrics and screening layers, we can greatly regulate the carrier density and improve the carrier mobility of few-layer MoS_2_ (almost fifteen times higher than on the SiO_2_ substrate at 5 K). Besides, the MoS_2_ field-effect transistors (FETs) using Bi_2_SiO_5_ as dielectrics can operate at 0.5 V, exhibiting a large *I*_on_/*I*_off_ (>10^8^), an ignorable hysteresis (~3 mV), low DIBL value (~5 mV/V) and low gate leakage current (~10^−13 ^A).

## Results

### Structure, CVD growth, and characterization of layered Bi_2_SiO_5_

**Fig. 1:**
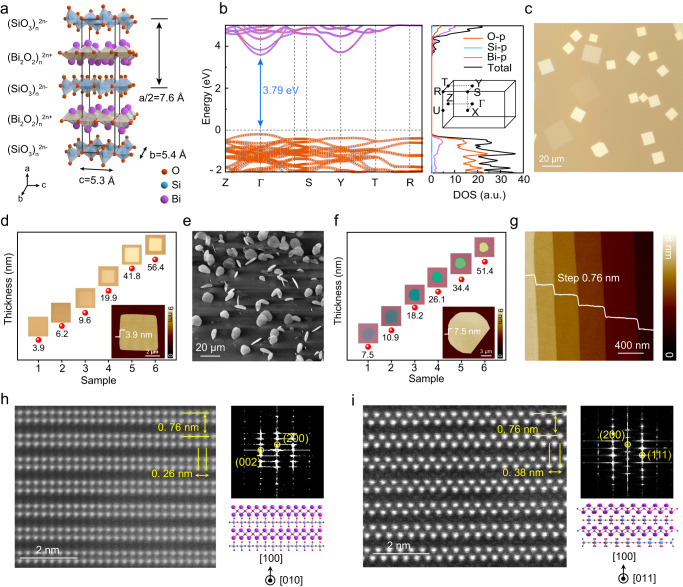
Structure, growth, and characterization of ultrathin Bi_2_SiO_5_ single crystals. **a** Crystal structure of Bi_2_SiO_5_ (*Cc*, *a* = 15.12 Å, *b* = 5.44 Å, *c* = 5.29 Å, *β* = 90.07°) with alternatively stacked [Bi_2_O_2_]_n_^2n+^ and [SiO_3_]_n_^2n−^ layers. **b** Calculated band structure and density of states (DOS) of Bi_2_SiO_5_ with a direct band gap of ~3.79 eV. The first Brillouin zone is inserted in the right panel. **c** Typical optical micrograph (OM) image of square Bi_2_SiO_5_ nanoplates showing an in-plane growth mode on mica substrate. **d** OM images of Bi_2_SiO_5_ nanoplates with thickness-dependent color contrasts on mica. The inset shows the typical atomic force microscope (AFM) image of an ultrathin Bi_2_SiO_5_ nanoplate with a thickness of 3.9 nm (5 layers) and an atomically smooth surface. **e** Scanning electron microscopy (SEM) image of 2D Bi_2_SiO_5_ crystals vertically grown on mica substrate. **f** Thickness-dependent color contrasts for Bi_2_SiO_5_ nanoplates transferred onto SiO_2_/Si substrate by a polymer-free mechanical pressing. The AFM image of a 7.5-nm-thick Bi_2_SiO_5_ nanoplate was inserted in **f**. **g** Typical AFM image of a terraced Bi_2_SiO_5_ nanoplates with a clear step of 0.76 nm. **h**, **i** Cross-sectional atomic-resolved high angle annular dark field (HAADF) images (left) and corresponding fast Fourier transform (FFT) diffraction spots (right) of chemical vapor deposition (CVD) grown Bi_2_SiO_5_ nanoplates taken along the zone axes of [010] (**h**) and [011] (**i**), respectively.

As shown in Fig. 1a, bismuth silicate Bi_2_SiO_5_ possesses a monoclinic lattice with *Cc* space group (quasi-orthogonal, *a* = 15.12 Å, *b* = 5.44 Å, *c* = 5.29 Å, *β* = 90.07°) and has an Aurivillius-type layered crystal structure with alternatively stacked [Bi_2_O_2_]_n_^2n+^ and [SiO_3_]_n_^2n-^ layers along the *a*-axis. The first-principle calculations were performed to investigate the band structure of layered Bi_2_SiO_5_. As shown in Fig. 1b, Bi_2_SiO_5_ exhibits a large direct band gap of ~3.79 eV, whose conduction band minimum (CBM) and valance band maximum (VBM) are both locate at Γ point of the first Brillouin zone and mainly originate from Bi-*p* and O-*p* orbits, respectively.

The essential prerequisite for using Bi_2_SiO_5_ as single-crystalline dielectrics with strong gate control is to achieve its growth of atomically thin films. However, the CVD growth of ultrathin Bi_2_SiO_5_ crystals remains a challenge yet. To our knowledge, SiO_2_ is chemically inert and has a very high melting point, which prevents it to be used as Si supplier during the CVD growth of Si-based compounds to some extent. Nevertheless, it’s well known that the fluorides will react with SiO_2_ to form volatile Si-based precursors. As a result, we developed a facile CVD method for the synthesis of Bi_2_SiO_5_ ultrathin crystals by using the BiF_3_ powders as Bi supplier and SiO_2_ powders or quartz boat as Si supplier (see Supplementary Fig. 1). With this method, ultrathin Bi_2_SiO_5_ crystals with various thicknesses can be readily obtained on mica substrates. As shown in Fig. 1c, d and Supplementary Fig. 2, at a relatively high growth temperature (~1023 K), Bi_2_SiO_5_ adopts a preferable in-plane growth mode on mica, which is widely observed in the CVD synthesis of layered materials, revealing a transparent square-like shape and atomically thin nature (down to monolayer, Supplementary Fig. 3). Normally, the in-plane growth mode will result in a large attaching area and strong bonding force between the epitaxial layer and the substrate, thereby adding difficulties for sample transfer and subsequent vdWs integration. Remarkably, the preferable growth mode of Bi_2_SiO_5_ on mica can be regulated from in-plane to out-of-plane growth modes just by lowering its growth temperature, which is similar to the case of Bi_2_O_2_Se^30^. As shown in Fig. 1e and Supplementary Fig. 2b, c, vertically grown ultrathin Bi_2_SiO_5_ crystals gradually emerge on mica at ~973 K, then dominates the CVD growth while further lowering the temperature to ~923 K. Unlike the case in Fig. 1c, vertically grown Bi_2_SiO_5_ crystals with thickness ranging from 7.5 to 50 nm can be easily transferred onto various substrates (such as SiO_2_/Si, Fig. 1e) by a polymer-free mechanical pressing (Supplementary Fig. 4), showing an atomically flat surface even after sample transfer (Fig. 1f). The transferable feature without unfavorable residual polymer contaminations makes free-standing Bi_2_SiO_5_ appealing for fabricating vdWs heterojunction device. Occasionally, the CVD-grown Bi_2_SiO_5_ nanoflakes revealed a terraced morphology with a step height of ~0.76 nm (Fig. 1g), consistent with the theoretical value for the monolayer step in Bi_2_SiO_5_. It’s worth noting that the CVD-grown Bi_2_SiO_5_ shows excellent air stability, whose surface morphology and roughness of Bi_2_SiO_5_ nanoplates remain almost the same when exposed to air for more than 7 months, which is an important metric for device fabrication as a high-*κ* gate dielectric (Supplementary Figs. 5, 6). More details about the comparison experiments conducted to understand how the reaction goes in the CVD growth of Bi_2_SiO_5_ and the possible reason for its in-plane and out-of-plane growth can be found in the discussion part of supporting information (Supplementary Figs. 7, 8).

The crystalline phase of as-grown samples was confirmed by transmission electron microscopy (TEM), Raman spectroscopy, and X-ray diffraction (XRD). As shown in Fig. 1h, i and Supplementary Fig. 9, we performed high-resolution TEM imaging along three-zone axes [namely (010), (011), and (100)], as well as corresponding fast Fourier transform (FFT) fringes. Based on the atomic-resolved cross-sectional TEM technique, the alternative stacking of [Bi_2_O_2_]_n_^2n+^ and the [SiO_3_]_n_^2n−^ layers with a layer space of 0.76 nm was observed. Besides, the averaged atomic ratio of Bi/Si was measured close to 2: 1 by energy dispersive spectroscopy (EDS, Supplementary Figs. 9, 10), which is consistent with the chemical formula of Bi_2_SiO_5_. The active modes at 70, 97, 149, 208, 298, 372, 433 cm^−1^ of the Raman spectra also matched well with monoclinic *Cc* phase of Bi_2_SiO_5_ (Supplementary Fig. 11)^35^. Additionally, the sharp XRD peaks, which can be assigned to (*h*00) crystal planes, further confirmed the high crystalline quality of the CVD-grown Bi_2_SiO_5_ nanoplates (Supplementary Fig. 11c).

### Dielectric properties of vertically grown Bi_2_SiO_5_ nanoplates

**Fig. 2:**
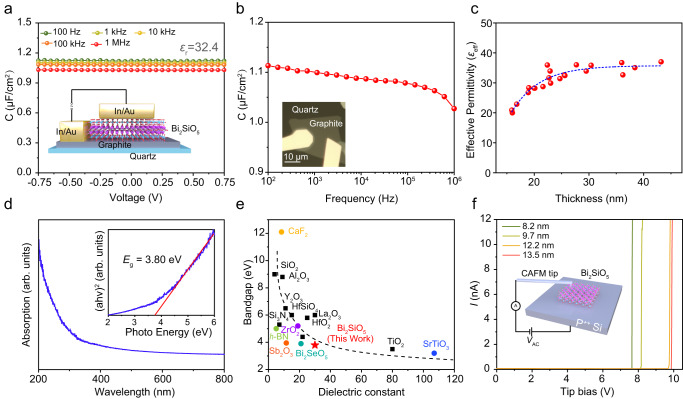
Dielectric constant, band gap, and breakdown field strength of CVD-grown Bi_2_SiO_5_ nanoflakes. **a** Typical Bias-dependent capacitance (*C*) measurements on CVD-grown Bi_2_SiO_5_ nanoflake with a common metal-insulator-metal (MIM) device configuration, where the thick graphite and In/Au metals serve as the bottom and top electrodes (inset), respectively. The dielectric constant (*ɛ*_r_) of Bi_2_SiO_5_ was estimated as ~32.4 at 100 Hz. **b** Corresponding frequency-dependent capacitance (*C*–*f*) characteristics of the MIM device, whose OM image is inset in **b**. **c** The thickness-dependent effective permittivity (*ε*_eff_) of Bi_2_SiO_5_ nanoflakes with a measuring frequency of 100 Hz. The dashed blue curve is a visual guide. **d** Ultraviolet-visible (UV-vis) absorption spectrum of CVD-grown Bi_2_SiO_5_ nanoflakes transferred onto quartz substrate with high coverage by mechanical pressing. The inset shows the fitting of its optical band gap (~3.8 eV) by Tauc’s law, where *a*, *h,* and *ν* are the absorption coefficient, Planck constant and frequency, respectively. **e** Energy band gap versus dielectric constant of representative dielectric materials in literature, showing the coexistence of high dielectric constant and large band gap in Bi_2_SiO_5_. The dashed line is a visual guide. **f** Thickness-dependent current-voltage curves of Bi_2_SiO_5_ nanoplates measured by C-AFM, showing a high breakdown field strength of 9.4 MV/cm (8.2 nm).

The transfer feasibility for vertically grown Bi_2_SiO_5_ nanoplates greatly facilitates the evaluation of their intrinsic properties, such as dielectric constant, band gap, and breakdown field strength. As shown in Fig. 2a, metal-insulator-metal (MIM) capacitors were fabricated on quartz substrates to extract the dielectric constant of Bi_2_SiO_5_ nanoflakes with varied thicknesses by capacitance-voltage (*C*–*V*) measurements. Here, the thick graphite and In/Au metals serve as the bottom and top electrodes, respectively. Thanks to high-*κ* nature, Bi_2_SiO_5_ nanoflake with a thickness of 25.6 nm demonstrated a very high capacitance density of 1.12 μF/cm^2^ at 100 Hz (Fig. 2a), revealing a slight decrease when the measuring frequency is up to 1 MHz. The corresponding capacitance-frequency (*C*–*f*) measurements showed the similar results (Fig. 2b). It is worth noting that the absolute capacitance value (>1 × 10^−12 ^F) measured is ~2~3 orders higher than the instrument’s offset and noise level (<1.5 × 10^−14 ^F, Supplementary Fig. 12). Based on the *C*–*V* and *C*–*f* data, we can estimate the effective permittivity (*ɛ*_eff_) of Bi_2_SiO_5_ to be ~32.4 at 100 Hz, which is preferable than commercial amorphous high-*κ* oxide such as Al_2_O_3_ (*k* = 7~9)^13,40,41^ and HfO_2_ (*k* = 13~17)^41,42^, and higher than most of reported vdWs single-crystalline dielectrics, such as *h*-BN (*k* = ~3.5)^43,44^, CaF_2_ (*k* = 8.4)^14^, Bi_2_SeO_5_ (*k* = 15.6)^18^, VOCl (*k* = 11.7)^45^ and ZrO_2_ (*k* = 8~19)^17^. Furthermore, we investigated the thickness-dependent *ɛ*_eff_ by fabricating MIM capacitors with different Bi_2_SiO_5_ thicknesses, from which we can extract very large *ɛ*_eff_ values of >30 in a wide thickness range (Fig. 2c). The gradual decrease of *ɛ*_eff_ while thinning down can be ascribed to the existence of interfacial “dead layer” in the MIM device, which is similar to other MIM devices^15,46–48^.

An ideal gate dielectric also needs a large band gap to inhibit the current leakage. Here, vertically grown Bi_2_SiO_5_ nanoflakes were directly transferred onto the polished quartz substrate by mechanical pressing for the ultraviolet-visible (UV-vis) absorption measurements. As shown in Fig. 2d, the optical band gap of CVD-grown Bi_2_SiO_5_ can be extracted as ~3.80 eV by Tauc’s law, which is consistent with the theoretical value (3.79 eV, Fig. 1b). To illustrate the metrics of Bi_2_SiO_5_ as a gate dielectric, the relationship between band gap and dielectric constant of representative gate dielectrics in literature was plotted in Fig. 2e, clearly indicating the coexistence of high dielectric constant and large band gap in Bi_2_SiO_5_. Moreover, we evaluated the breakdown field strength (*E*_bd_) of the Bi_2_SiO_5_ nanoplates by conductive atomic force microscope (C-AFM) measurements on p^++^ Si. As shown in Fig. 2f, the CVD-grown Bi_2_SiO_5_ showed thickness-dependent breakdown field strength ranging from 7.2 (13.5 nm) to 9.4 (8.2 nm) MV cm^−1^, which is close to that of dielectrics in the silicon industry such as Al_2_O_3_^49^ and HfO_2_^50^ but owns higher dielectric constant. We should emphasize that the C-AFM is a very local and microscopic tool to measure the breakdown voltage of a dielectric insulator, which may be not the same as the global one determined by MIM device. For example, the breakdown experiments based on the MIM devices gave a breakdown field strength of 3~5 MV/cm instead while varying the thickness of Bi_2_SiO_5_ from 10.1 to 21.4 nm (Supplementary Fig. 13).

**Fig. 3:**
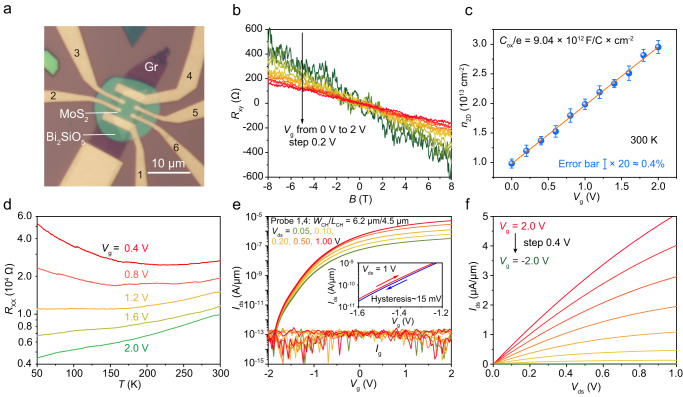
MoS_2_ Hall-bar device with the vertically grown ultrathin Bi_2_SiO_5_ nanoflake as the high-*κ* back-gate dielectrics. **a** OM image of an as-fabricated MoS_2_ (~2.7 nm) Hall-bar device on SiO_x_/Si substrate, in which a thin flake of Bi_2_SiO_5_ (~22 nm) and multi-layer graphene were adopted as gate dielectric and back-gate electrode, respectively. **b** Hall resistance (*R*_*xy*_) as a function of magnetic field (*B*) under various gate voltages (*V*_g_) from 0 to 2 V at 300 K. **c** The extracted *V*_g_-dependent sheet carrier density (*n*_2D_), in which a high dielectric constant (*ε*_r_) of ~36 was derived by linear fitting. The Error bars are based on the standard deviations of slope fitting on *R*_xy_-*B* curves. **d** Longitudinal resistance (*R*_xx_) as a function of temperature (*T*) under different *V*_g_ from 0.4 to 2.0 V, showing a gate-induced insulator-to-metal transition. **e** Two-probe transfer curves of the back-gated MoS_2_/Bi_2_SiO_5_ field-effect transistor (FET) measured by defining the probes of 1 and 4 as source and drain terminals, showing *I*_on_/*I*_off_ > 10^8^, *SS* ~ 64 mV/decade. The inset shows a small hysteresis of ~15 mV. The extracted two-terminal field-effect mobility of the device is 18.6 cm^2^ V^−1^ s^−1^. **f** The corresponding output curves measured in a *V*_g_ range from 2 to −2 V.

**Fig. 4:**
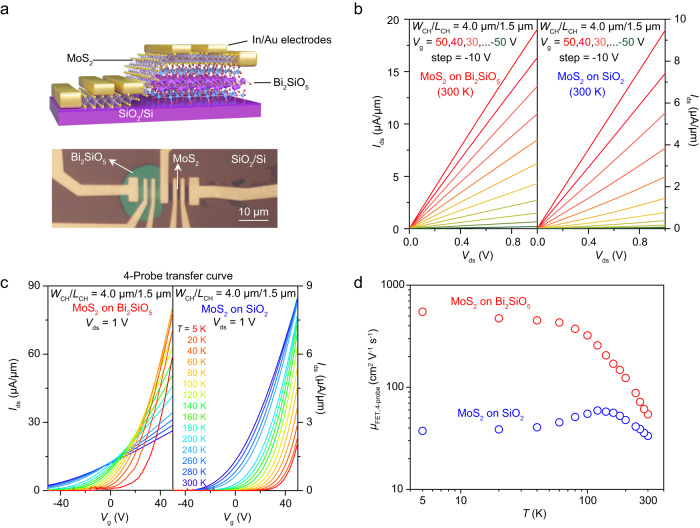
Dielectric screening and mobility enhancement effects of CVD-grown Bi_2_SiO_5_ nanoflakes as the high-*κ* substrates. **a** Schematic illustration and OM image of back-gate MoS_2_ four-probe FETs device. **b** Linear output curves (*I*_ds_–*V*_ds_) of MoS_2_ FETs on Bi_2_SiO_5_ (left) and on SiO_2_ substrate (right) measured at 300 K. **c** The 4-probe transfer curves of MoS_2_ FETs measured at different temperatures (5~300 K) on Bi_2_SiO_5_ (left) and SiO_2_ (right) substrates, respectively. **d** The extracted temperature-dependent 4-probe FET mobility (*μ*_FET,4-probe_) of MoS_2_ on Bi_2_SiO_5_ (red) and SiO_2_ (blue) substrates.

**Fig. 5:**
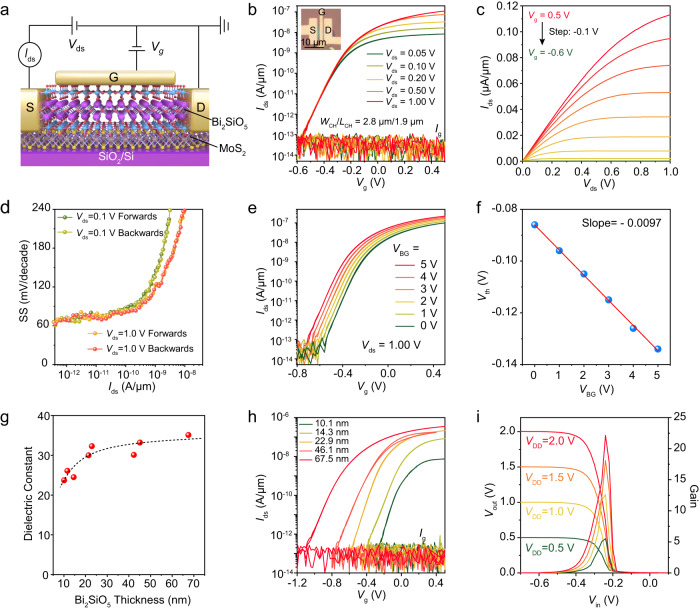
Hysteresis-free MoS_2_ FETs and low-power NMOS inverters using Bi_2_SiO_5_ as high-*κ* top-gate dielectrics. **a** Schematic illustration of the top-gated MoS_2_ FETs on SiO_2_/Si substrate with Bi_2_SiO_5_ as the top-gate dielectric. **b** Typical dual-sweep transfer curves of the MoS_2_/Bi_2_SiO_5_ FET measured under different *V*_ds_ from 0.05 to 1 V, showing an ideal *SS* value of ~62 mV/decade and ignorable gate hysteresis. The Insert is the OM image of a fabricated MoS_2_ FET with Bi_2_SiO_5_ as the gate dielectric. **c** Corresponding output characteristics (*I*_ds_–*V*_ds_ curves) of the device measured by varying the *V*_g_ from 0.5 to −0.6 V with a 0.1 V step. **d** Extracted *SS* value versus *I*_*ds*_ characteristics of the device in **b**, showing a low SS value (<70 mV/decade) for a wide *I*_ds_ range. **e** Dual gated transfer curves of the MoS_2_/ Bi_2_SiO_5_ FET under different back-gate voltages (*V*_BG_) from 5 to 0 V. **f** The extracted threshold voltage *V*_th_ from **e** as a function of *V*_BG_. The linear fitting yields a slope of −0.0097 and a high dielectric constant of ~32.3. **g** The thickness-dependent dielectric constant of Bi_2_SiO_5_ extracted by dual-gate measurement on Bi_2_SiO_5_/MoS_2_/SiO_2_/Si FET. The dotted curve is a visual guide. **h** Transfer curves of the MoS_2_ FETs with different thickness Bi_2_SiO_5_ as the top-gate dielectrics, showing a trend of smaller *V*_th_ for thinner Bi_2_SiO_5_ thickness. The *V*_ds_ is 0.5 *V*, and no *V*_BG_ is applied. **i** Measured output voltage (*V*_out_) and gain as a function of input voltage (*V*_in_) of an NMOS inverter based on two MoS_2_/ Bi_2_SiO_5_ FETs under different supply voltage (*V*_dd_) from 2 *V* to 0.5 V.

The features of easy-to-transfer, ultra-flat surface, high dielectric constant, large band gap, and breakdown field strength make Bi_2_SiO_5_ highly competitive as gate dielectrics and high-*κ* substrates for dielectric screening. Here, we combine Bi_2_SiO_5_ with mechanically exfoliated few-layer MoS_2_ to construct Bi_2_SiO_5_/MoS_2_ FETs for demonstrating its advantages as back-gate dielectrics (Fig. 3), dielectric screening substrate (Fig. 4) and top-gate dielectrics (Fig. 5).

### Vertically grown Bi_2_SiO_5_ as back-gate dielectric

The small contact area and weak interaction between the vertically grown Bi_2_SiO_5_ and the mica substrate make it compatible with the well-developed aligned transfer method (For details, see Supplementary Fig. 14)^19,20^, allowing us to fabricate complex vdWs heterojunction devices by layer-by-layer stacking. Figure 3a showed the OM image of an as-fabricated MoS_2_ Hall-bar device using Bi_2_SiO_5_ as the back-gate dielectric and multi-layer graphene as a back-gate electrode. Gated Hall measurement is a powerful tool to get a series of key parameters, such as the dielectric constant of the gate dielectric, carrier density, and Hall mobility of the channel semiconductor. Besides, it also enables us to get the transfer and output characteristics by defining two out of six electrodes as source and drain. Figure 3b showed Hall resistance (*R*_xy_) as a function of magnetic field (*B*) under different gate voltages (*V*_g_) at room temperature, from which we can extract the *V*_g_-dependent sheet carrier densities (*n*_2D_). By linear fitting the curve of *n*_2D_-*V*_g_ (Fig. 3c), the estimated slope equals the *C*_ox_/e, where *C*_ox_ is capacitance density and e is the elementary charge. The *C*_ox_ for a 22-nm-thick Bi_2_SiO_5_ can be as high as 1.45 μF/cm^2^, suggesting a very high dielectric constant (*ɛ*_r_ ~ 36), which is consistent with the value extracted by *C*–*V* measurements in a reasonable accuracy. On the other hand, very large capacitance density suggests that we can greatly regulate the carrier density and electrical behavior of the MoS_2_ semiconductor. As confirmed by the room-temperature Hall measurements (Fig. 3c), a very high carrier density of 1.83 × 10^13 ^cm^−2^ can be doped into the channel by applying a *V*_g_ of 2 V on Bi_2_SiO_5_ dielectrics. To this end, the temperature-dependent longitudinal resistance (*R*_xx_-*T*) behavior of MoS_2_ can be greatly regulated from insulating to metallic one when sweeping the *V*_g_ from 0.4 to 2 V (Fig. 3d), indicating the great potential of Bi_2_SiO_5_ dielectric as a powerful tool for physical state regulation.

To examine whether ultrathin Bi_2_SiO_5_ can serve as excellent back-gate dielectrics of a FET, we define the electrodes #1, #4 and graphene as source, drain, and gate electrodes, respectively. The dual-sweep transfer curves of MoS_2_/Bi_2_SiO_5_/Gr FET were presented in Fig. 3e, showing a large *I*_on_/*I*_off_ of >10^8^, and a small SS value of ~64 mV/decade. Considering the relatively complex device fabrication process (Supplementary Fig. 14), chemical residuals will inevitably remain at the interfaces between the graphene, Bi_2_SiO_5_ and MoS_2_, which are detrimental to the device performance. Nevertheless, the FET still exhibited small hysteresis of ~15 mV, which is comparable to the value obtained after interface optimization^51^. Thanks to the Ohmic contact formed by In/Au electrodes (Fig. 3f), two-terminal field-effect mobility of the device can be as high as 18.6 cm^2^ V^−1^ s^−1^ by linear fitting the transfer curve, which is comparable to the Hall mobility measured at room temperature (23.7 cm^2^ V^−1^ s^−1^ at *V*_g_ = 2 V, Supplementary Fig. 15). Notably, Bi_2_SiO_5_ was also verified as an excellent gate insulator while shrinking the channel length of MoS_2_ FETs down to 100 nm and even 30 nm (Supplementary Figs. 16, 17).

### Vertically grown Bi_2_SiO_5_ as high-*κ* screening layer

Dangling bonds on SiO_2_/Si substrate usually act as the scattering sites of Coulomb impurities (CI) to decrease the mobility of a semiconductor^52–54^. Theoretically speaking, using an *h*-BN-like high-*κ* substrate free of dangling bonds can effectively enhance its mobility due to reduced CI scatterings and excellent dielectric screening. Figure 4a shows the scheme and OM image of as-fabricated 4-probe MoS_2_ FETs on top of Bi_2_SiO_5_ and SiO_2_/Si substrates. To avoid the influence of sample quality variation, one MoS_2_ sample (4.3 nm) was simultaneously placed on Bi_2_SiO_5_ (31.9 nm) and SiO_2_ substrates for comparison. As demonstrated in Fig. 4b, the *V*_g_-dependent two-probe *I*–*V* curves (output characteristics) were measured at 300 K, showing a linear behavior over a large voltage window, confirming the Ohmic contact formed by In/Au electrodes. It’s worth noting that the on-state current (*I*_on_) of MoS_2_ on Bi_2_SiO_5_ is about twice as the one on SiO_2_, suggesting a higher mobility of MoS_2_ on Bi_2_SiO_5_ substrate. To illuminate the influence of contact resistance, 4-probe transfer curves were measured among a temperature range of 5~300 K (Fig. 4c). By linear fitting the transfer curves, we can extract the 4-probe FET mobility (*μ*_FET,4-probe_) as a function of temperature (Fig. 4d). Among the whole temperature range of 300~5 K, the carrier mobility of MoS_2_ on Bi_2_SiO_5_ is significantly higher than that on SiO_2_/Si substrate, which can be further confirmed by plotting the *I*_ds_ as a function of a normalized *V*_g_ by threshold voltage (*V*_g_-*V*_th_, Supplementary Fig. 18). Particularly, the mobility of MoS_2_ on Bi_2_SiO_5_ at 5 K is as high as 549.3 cm^2^ V^−1^ s^−1^, which is almost fifteen times higher than the value of MoS_2_ on SiO_2_/Si (~37.4 cm^2^ V^−1^ s^−1^). Moreover, the carrier mobility of MoS_2_ on Bi_2_SiO_5_ and SiO_2_/Si showed totally different temperature dependence. For MoS_2_/Bi_2_SiO_5_, its carrier mobility increased monotonously upon cooling down, suggesting the phonon scattering dominated the whole transport events even at low temperature. In contrast, the carrier mobility of MoS_2_ on SiO_2_/Si substrate increased first while cooling down to ~150 K, but gradually decreased upon further cooling. This *T*-dependent mobility can be well explained by enhanced charge impurities scattering at low temperature in MoS_2_/SiO_2_ interface. In a word, using Bi_2_SiO_5_ as a substrate greatly improve the performance of the MoS_2_ FET to get a higher mobility, which can be attributed to the suppressed CI scatterings in the high-*κ* surroundings and ideal dielectric/semiconductor interface.

### Vertically grown Bi_2_SiO_5_ as the top-gate dielectric

Top-gate FET is a widely used device configuration in practical applications. In this part, we fabricated Bi_2_SiO_5_/MoS_2_ top-gate FETs to prove the feasibility of Bi_2_SiO_5_ as top-gate dielectric layer in hysteresis-free low-power transistors (Supplementary Fig. 19). As shown in Fig. 5a, the Bi_2_SiO_5_/MoS_2_ top-gate FET was placed on the SiO_2_/Si substrate, which can also serve as the back-gate dielectric and electrode when necessary. Figure 5b showed the typical dual-sweep transfer curves of a 22.9-nm-thick Bi_2_SiO_5_-based MoS_2_ FET measured under different *V*_ds_ from 0.05 to 1 V. Owing to the excellent gate tunability, the transistor can be effectively turned on and off by applying a *V*_g_ within the range of −0.6~0.5 V, showing a large *I*_on_/*I*_off_ of >10^6^ and a low gate leakage current of 10^−13 ^A (approaching the detection limit). Furthermore, the ideal SS value (~62 mV/decade), ignorable gate hysteresis (~3 mV), and low drain-induced barrier lowering (DIBL, ~5 mV/V) suggest the perfect interface was formed at MoS_2_/Bi_2_SiO_5_ interface (Supplementary Fig. 20). The MoS_2_ transistor showed a linear *I*_ds_-*V*_ds_ curve at low *V*_ds_ region, then gradually saturated at high *V*_ds_ region (Fig. 5c). Particularly, the SS remains low (<70 mV/decade) for different *I*_ds_ of several orders of magnitude for both forward and reverse top-gate sweeping (Fig. 5d). More importantly, the top-gate Bi_2_SiO_5_/MoS_2_ short-channel FET, whose channel length was defined by the gap distance between two graphene electrodes (~180 nm), still showed a small DIBL value of ~22 mV/V and SS value of ~79 mV/decade (Supplementary Fig. 21).

One step further, nearly hysteresis-free transfer curves were preserved in a dual-gate FET configuration, in which a back-gate voltage (*V*_BG_) was employed to modulate the threshold voltage (*V*_th_) of the device. As a result, the top-gate transfer curves gradually shifted as the *V*_BG_ varied from 5 to 0 V (Fig. 5e and Supplementary Fig. 22). By linear fitting the *V*_th_ as a function of *V*_BG_, we can extract a slope of −0.0097, which equals to the ratio of the bottom-gate to top-gate capacitance, namely *C*(SiO_2_)/*C*(Bi_2_SiO_5_), when the parallel-plate capacitor model is assumed for both top and bottom gates^16,55^. For a 285 nm SiO_2_ dielectric (*ɛ*_r_ = 3.9), its capacitance can be calculated as 0.0121 μF/cm^2^. In this case, the capacitance and dielectric constant for a 22.9-nm-thick Bi_2_SiO_5_ were derived as 1.25 μF/cm^2^ and 32.3, both of which matched well with the value obtained by *C*–*V* (Fig. 2a) and gated Hall measurements (Fig. 3c). Next, the interface trap density *D*_it_ was extracted based on the following equation:^15^

1 $${SS}={{{{{\rm{ln}}}}}}\left(10\right)\frac{{k}_{B}T}{q}\left(1+\frac{q{D}_{{it}}}{{C}_{{ox}}}\right)$$

where *SS* is the subthreshold swing, *k*_B_ is Boltzmann constant, *T* is absolute temperature, *q* is the elementary charge, *C*_ox_ is the gate capacitance obtained from MOS capacitance measurements. As a result, a low *D*_it_ value of 2.88 × 10^11 ^cm^−2^/eV was extracted, verifying the high quality of the vdWs interface.

Moreover, the dielectric constant of Bi_2_SiO_5_, extracted by dual-gate transfer measurements, also showed similar thickness dependence with the *C*–*V* results (Fig. 5g). Figure 5h plotted the dual-sweep transfer curves of MoS_2_ FETs with different Bi_2_SiO_5_ thicknesses (10.1~67.5 nm). Apparently, smaller *V*_g_ is needed to switch the transistor on and off when a thinner Bi_2_SiO_5_ dielectric is used. We should emphasize that the EOT value for a 10.1 nm Bi_2_SiO_5_ is as small as 1.3 nm, but its gate leakage current is still on the order of 10^−13 ^A, signifying substantial room space for further scaling of EOT and great potential applications in low-power devices. The *I*_on_ of MoS_2_ FETs in Fig. 5h seems to decrease with decreasing the thickness of Bi_2_SiO_5_, which may originate from the contact issues existing in the MoS_2_ FETs with the thin Bi_2_SiO_5_ as gate insulators. However, we should emphasize that, the *I*_on_ of ultrathin Bi_2_SiO_5_-gated MoS_2_ FETs can be greatly improved by optimizing the device fabrication process. As shown in Supplementary Figs. S23–25, the *I*_on_ of another 10-nm-thick Bi_2_SiO_5_-gated MoS_2_ can be similar to the value of the thick Bi_2_SiO_5_-gated FETs (0.11 μA/μm, Fig. 5b). It is worth noting that the *I*_on_ is greatly limited by the remaining ungated channel in the top-gate device configuration. Additionally, as confirmed by the dual-sweep transfer curves, a nearly ideal SS value and a low normalized gate hysteresis can indeed be obtained in the Bi_2_SiO_5_ gated MoS_2_ FET.

The low operating voltage is essential to fabricate low-power logic circuits. As demonstrated in Fig. 5i, we used two n-type transistors as the load and driver terminal to construct 2D inverter with a high voltage gain of 22.0. The inverter can demonstrate the logic state 0 and 1 within ±0.5 V, showing a low dynamic power consumption of <0.7 nW (Supplementary Fig. 26).

In summary, our work achieved the direct CVD growth of ultrathin free-standing high*-k* single-crystalline dielectrics, which is much more efficient than traditional mechanical exfoliation. The vertically grown Bi_2_SiO_5_ 2D crystals present the metrics for a gate dielectric, as evidenced by the coexistence of high dielectric constant, large band gap, high breakdown field strength, as well as the characteristic of easy transfer by facile polymer-free mechanical pressing. These features make Bi_2_SiO_5_ attractive as an inert vdWs substrate superior to *h*-BN for exploring exotic transport properties under reduced interfacial scatterings and stronger gate control, as well as for fabricating hysteresis-free 2D transistors with scaled supply voltage.

## Methods

### CVD growth of ultrathin Bi_2_SiO_5_ nanoplates on mica substrate

2D Bi_2_SiO_5_ crystals were synthesized inside a homemade CVD system equipped with a single heating zone tube furnace and 30 mm diameter quartz tube. Typically, the BiF_3_ powders (purity 99.999%, Macklin) were placed in an empty quartz boat or on top of the SiO_2_ powders located in the heating center, and the freshly cleaved fluorophlogopite mica substrates were placed above the quartz boat. The heating temperature of the source was 600–750 °C and the growth time was 20 min. The 50 s.c.c.m Ar and 5 s.c.c.m mixed Ar/O_2_ (1‰) gas were introduced into the CVD system as carrier gas. The system pressure was kept constant as 760 Torr during the whole growth process.

### Characterization of CVD-grown ultrathin Bi_2_SiO_5_ single-crystalline dielectric

The morphologies of as-synthesized in-plane and free-standing 2D Bi_2_SiO_5_ nanoplates were characterized by optical microscopy (Olympus BX53), scanning electron microscopy (SEM, JSM-7800F) and atomic force microscope (AFM, Bruker dimension icon). With a polymer-free method of mechanical pressing, vertically grown Bi_2_SiO_5_ nanoflakes were transferred onto Cu grid, glass, and optical quartz substrates to perform the characterizations of transmission electron microscopy (TEM, JEM 2800), X-ray diffraction (XRD, Rigaku Smart Lab 30 KW) and absorption spectrum (SHIMADZU, UV-2600), respectively. The Raman spectroscopy was measured on WITec alpha300R with a laser of 532 nm. The cross-sectional TEM samples of in-plane and vertically grown Bi_2_SiO_5_ nanoflakes were both prepared by using a focused ion/electron dual beam system (FEI, Helios 5 CX). All cross-sectional scanning transmission electron microscopy (STEM) imaging was obtained on an aberration-corrected TEM operating at 300 kV (FEI Titan cubed Themis G2 300). The breakdown field strength of Bi_2_SiO_5_ was measured with the C-AFM module of Bruker Dimension Icon.

### First-principles calculations

The structural relaxation of Bi_2_SiO_5_ is performed within the framework of density function theory (DFT) using the projector augmented wave pseudopotential and the Perdew–Burke–Ernzerhof exchange-correlation functional as implemented in the VASP. The energy cutoff for the plane-wave expansion is set to 500 eV, and a Monkhorst–Pack k-mesh of 3 × 9 × 9 is used in the Brillouin zone. The energy convergence threshold is 10^−6 ^eV and the force 10^−3 ^eV Å^−1^ in the structural optimization. In order to overcome the underestimation of energy gap from the generalized gradient approximation (GGA), we use the method of modified Becke–Johnson potential (mBJ) to calculate the electronic structure.

### Device fabrication

To illuminate the possible capacitance coupling, the Bi_2_SiO_5_-based metal-insulator-metal (MIM) capacitors were fabricated on quartz substrates rather than SiO_2_/Si substrates. First, the thick graphite was exfoliated onto the quartz substrate as bottom electrodes for its ultrasmooth surface. Next, the vertically grown Bi_2_SiO_5_ nanoflakes were directly picked up from the mica substrate by a polypropylene carbonate/polydimethylsiloxane (PPC/PDMS) stamp, followed by aligned transfer onto the specific graphite bottom electrode by a high-precision transfer platform. Subsequently, the standard electron-beam lithography (EBL) process and thermal evaporation were used to pattern the top electrodes ((In/Au, 5/40 nm).

For fabricating the back-gated MoS_2_ Hall-bar device using graphite as bottom electrodes, the detailed process was listed as follows. First, SiO_2_/Si substrates (285 nm SiO_2_) were pretreated with O_2_ plasma (Diener Pico plasma cleaner) for 5 min at a power of 50 W. Next, few-layer MoS_2_ and graphite were exfoliated onto different SiO_2_/Si substrates. With the help of the PPC/PDMS stamp and high-precision transfer platform, the free-standing Bi_2_SiO_5_ and MoS_2_ nanoflakes were sequentially stacked on top of the graphite. The six-terminal electrode legs for the Hall-bar and the bottom metal electrodes were simultaneously written by one-step EBL and following thermal metal deposition (In/Au, 5/40 nm). The device fabrication process of 4-terminal MoS_2_ FET includes the following parts: (1) transfer the Bi_2_SiO_5_ onto SiO_2_/Si substrate by mechanical pressing; (2) place part of the few-layer MoS_2_ on top of the Bi_2_SiO_5_ nanosheet; (3) EBL and metal deposition (In/Au, 5/40 nm).

For the top-gated MoS_2_ device, few-layer MoS_2_ nanosheets were exfoliated onto SiO_2_/Si substrate, followed by stacking Bi_2_SiO_5_ nanoflakes in the middle of the MoS_2_ nanosheets as the gate dielectrics. The source, drain, and top-gate electrodes of MoS_2_ FETs were patterned together with one-step EBL process and thermal metal evaporation (In/Au, 5/40 nm).

### Electrical transport measurements

2-probe electrical properties of the back-gate and top-gate MoS_2_ FETs, including the Figs. 3e, f, 4b, 5b, c, e, h, were carried out by a semiconductor analyzer (FS-Pro) in a shielded vacuum chamber (<0.1 Torr) at room temperature, whose noise level is ~1 × 10^−13 ^A within the voltage range of ±2 V. The 4-probe transfer curves and gated 4-probe measurements (such as gated *R*_xx_-*T* and Hall data), including Figs. 3b, d, 4c, were carried out in a Physical Properties Measurement Systems (PPMS-9T, Quantum Design) equipped with a homemade electrical measurement system, which is composed of 2 Keithley 2400, and 2 Keithley 2182 A nanovoltmeter and has a noise level of ~10^−10 ^A within the voltage range of ± 2 V. The *C*–*V* and *C*–*f* measurements were carried out on a FS336 LCR Meter.

## Supplementary information

Supplementary Information

Peer Review File

## Data Availability

Relevant data supporting the key findings of this study are available within the article and the Supplementary Information file. All raw data generated during the current study are available from the corresponding authors upon request.

## References

[1] Liu Y (2021). Promises and prospects of two-dimensional transistors. Nature.

[2] Jiang J (2023). Ballistic two-dimensional InSe transistors. Nature.

[3] Tan C (2023). 2D fin field-effect transistors integrated with epitaxial high-k gate oxide. Nature.

[4] Desai SB (2016). MoS_2_ transistors with 1-nanometer gate lengths. Science.

[5] Kang K (2015). High-mobility three-atom-thick semiconducting films with wafer-scale homogeneity. Nature.

[6] Das S (2021). Transistors based on two-dimensional materials for future integrated circuits. Nat. Electron..

[7] Wang SY (2022). Two-dimensional devices and integration towards the silicon lines. Nat. Mater..

[8] Yang SJ (2022). Gate dielectrics integration for 2D electronics: challenges, advances, and oulook. Adv. Mater..

[9] Lin YC (2023). Dielectric material technologies for 2-D semiconductor transistor scaling. IEEE Trans. Electron Devices.

[10] Illarionov YY (2020). Insulators for 2D nanoelectronics: the gap to bridge. Nat. Commun..

[11] Kim HG (2017). Atomic layer deposition on 2D materials. Chem. Mater..

[12] Xiao MM (2017). Atomic-layer-deposition growth of an ultrathin HfO_2_ film on graphene. ACS Appl. Mater. Interfaces.

[13] Li N (2019). Atomic layer deposition of Al_2_O_3_ directly on 2D materials for high-performance electronics. Adv. Mater. Interfaces.

[14] Illarionov YY (2019). Ultrathin calcium fluoride insulators for two-dimensional field-effect transistors. Nat. Electron..

[15] Huang JK (2022). High-k perovskite membranes as insulators for two-dimensional transistors. Nature.

[16] Yang AJ (2022). Van der Waals integration of high-k perovskite oxides and two-dimensional semiconductors. Nat. Electron..

[17] Jin, Y. et al. Controllable oxidation of ZrS_2_ to prepare high-k, single-crystal m-ZrO_2_ for 2D electronics. *Adv. Mater*. **35**, 2212079 (2023). 10.1002/adma.202212079 36815429

[18] Zhang, C. C. et al. Single-crystalline van der Waals layered dielectric with high dielectric constant. *Nat. Mater.* **22**, 832–837 (2023). 10.1038/s41563-023-01502-7 36894772

[19] Dean CR (2010). Boron nitride substrates for high-quality graphene electronics. Nat. Nanotechnol..

[20] Wang L (2013). One-dimensional electrical contact to a two-dimensional material. Science.

[21] Bandurin DA (2017). High electron mobility, quantum Hall effect and anomalous optical response in atomically thin InSe. Nat. Nanotechnol..

[22] Wu SF (2018). Observation of the quantum spin Hall effect up to 100 kelvin in a monolayer crystal. Science.

[23] Yang FY (2018). Quantum hall effect in electron-doped black phosphorus field-effect transistors. Nano Lett..

[24] Naclerio AE (2023). A review of scalable hexagonal boron nitride (h-BN) synthesis for present and future applications. Adv. Mater..

[25] Liu KL (2021). A wafer-scale van der Waals dielectric made from an inorganic molecular crystal film. Nat. Electron..

[26] Jin YY (2020). Growth of large-scale two-dimensional insulator Na_2_Ta_4_O_11_ through chemical vapor deposition. J. Semicond..

[27] Peng J (2021). Inorganic low k cage-molecular crystals. Nano Lett..

[28] Zhu CY (2022). 2D indium phosphorus sulfide (In_2_P_3_S_9_): an emerging van der Waals high-k dielectrics. Small.

[29] Han MJ (2022). Continuously tunable ferroelectric domain width down to the single-atomic limit in bismuth tellurite. Nat. Commun..

[30] Hong CY (2020). Inclined ultrathin Bi_2_O_2_Se films: a building block for functional van der Waals heterostructures. ACS Nano.

[31] Tan CW (2022). Strain-free layered semiconductors for 2D transistors with on-state current density exceeding 1.3 mA/μm^-1^. Nano Lett..

[32] Park J (2016). Tetrahedral tilting and ferroelectricity in Bi_2_AO_5_ (A=Si, Ge) from first principles calculations. J. Solid State Chem..

[33] Wu YT (2017). Preparation and fluorescence property of pure Bi_2_SiO_5_ powders by Pechini sol-gel method. Mater. Manuf. Process..

[34] Liu D (2018). Constructing a novel Bi_2_SiO_5_/BiPO_4_ heterostructure with extended light response range and enhanced photocatalytic performance. Appl. Catal. B.

[35] Depablos-Rivera O (2019). Synthesis of Bi_2_SiO_5_ thin films by confocal dual magnetron sputtering-annealing route. Thin Solid Films.

[36] Taniguchi H (2013). Ferroelectricity driven by twisting of silicate tetrahedral chains. Angew. Chem. Int. Ed..

[37] Kodera M (2019). Ferroelectric properties of epitaxial Bi_2_SiO_5_ thin films grown on SrTiO_3_ substrates with various orientations. Jpn. J. Appl. Phys..

[38] Kijima T (1998). Preparation of Bi_4_Ti_3_O_12_ thin film on Si(100) substrate using Bi_2_SiO_5_ buffer layer and its electric characterization. Jpn. J. Appl. Phys..

[39] Sakamoto K (2021). Fabrication of bismuth silicate Bi_2_SiO_5_ ceramics as a potential high-temperature dielectric material. J. Mater. Sci..

[40] Fisichella G (2017). Interface electrical properties of Al_2_O_3_ thin films on graphene obtained by atomic layer deposition with an in situ seedlike layer. ACS Appl. Mater. Interfaces.

[41] Chang YC (2011). Atomic-layer-deposited Al_2_O_3_ and HfO_2_ on GaN: a comparative study on interfaces and electrical characteristics. Microelectron. Eng..

[42] Mohanty S (2021). Investigation and optimization of HfO_2_ gate dielectric on N-polar GaN: Impact of surface treatments, deposition, and annealing conditions. Appl. Phys. Lett..

[43] Ahmed F (2018). Dielectric dispersion and high field response of multilayer hexagonal boron nitride. Adv. Funct. Mater..

[44] Kim KK (2012). Synthesis and characterization of hexagonal boron nitride film as a dielectric layer for graphene devices. ACS Nano.

[45] Zhu W (2021). Ternary VOCl single-crystal as efficient gate dielectric for 2D field-effect transistors. 2D Mater..

[46] Muller DA (1999). The electronic structure at the atomic scale of ultrathin gate oxides. Nature.

[47] Stengel M (2006). Origin of the dielectric dead layer in nanoscale capacitors. Nature.

[48] Lu Z (2023). Wafer-scale high-κ dielectrics for two-dimensional circuits via van der Waals integration. Nat. Commun..

[49] Lin HC (2005). Leakage current and breakdown electric-field studies on ultrathin atomic-layer-deposited Al_2_O_3_ on GaAs. Appl. Phys. Lett..

[50] Sire C (2007). Statistics of electrical breakdown field in HfO_2_ and SiO_2_ films from millimeter to nanometer length scales. Appl. Phys. Lett..

[51] Luo PF (2022). Molybdenum disulfide transistors with enlarged van der Waals gaps at their dielectric interface via oxygen accumulation. Nat. Electron..

[52] Yu ZH (2016). Realization of room-temperature phonon-limited carrier transport in monolayer MoS_2_ by dielectric and carrier screening. Adv. Mater..

[53] Joo MK (2017). Understanding coulomb scattering mechanism in monolayer MoS_2_ channel in the presence of h-BN buffer layer. ACS Appl. Mater. Interfaces.

[54] Cui X (2015). Multi-terminal transport measurements of MoS_2_ using a van der Waals heterostructure device platform. Nat. Nanotechnol..

[55] Li WS (2019). Uniform and ultrathin high-k gate dielectrics for two-dimensional electronic devices. Nat. Electron..

