# Supplementary material for: Vertically grown ultrathin Bi2SiO5 as high-κ single-crystalline gate dielectric
Source: Nat Commun. 2023 Jul 21;14:4406. doi: 10.1038/s41467-023-40123-1 (PMC10361963; doi:10.1038/s41467-023-40123-1)
Supplement: Supplementary file 1 — Supplementary Information [file 41467_2023_40123_MOESM1_ESM.docx]

Supplementary Materials for

**Vertically grown ultrathin Bi_2_SiO_5_ as high-*κ* single-crystalline gate dielectric**

Jiabiao Chen^1^, Zhaochao Liu^1^, Xinyue Dong^1^, Zhansheng Gao^1^, Yuxuan Lin^1^, Yuyu He^1^, Yingnan Duan^1^, Tonghuai Cheng^1^, Zhengyang Zhou^2^, Huixia Fu^3^, Feng Luo^1^, Jinxiong Wu^1,*^

*^1^Tianjin Key Lab for Rare Earth Materials and Applications, Center for Rare Earth and Inorganic Functional Materials, Smart Sensor Interdisciplinary Science Center, School of Materials Science and Engineering, Nankai University, Tianjin 300350, China*

*^2^State Key Laboratory of High Performance Ceramics and Superfine Microstructure, Shanghai Institute of Ceramics, Chinese Academy of Sciences, Shanghai 200093, China.*

*^3^Center of Quantum Materials and Devices & College of Physics, Chongqing University, Chongqing 401331, China*

**Corresponding author. Email:* [*jxwu@nankai.edu.cn*](mailto:jxwu@nankai.edu.cn)

**Supplementary Note 1: CVD growth, transfer and air stability of Bi_2_SiO_5_**


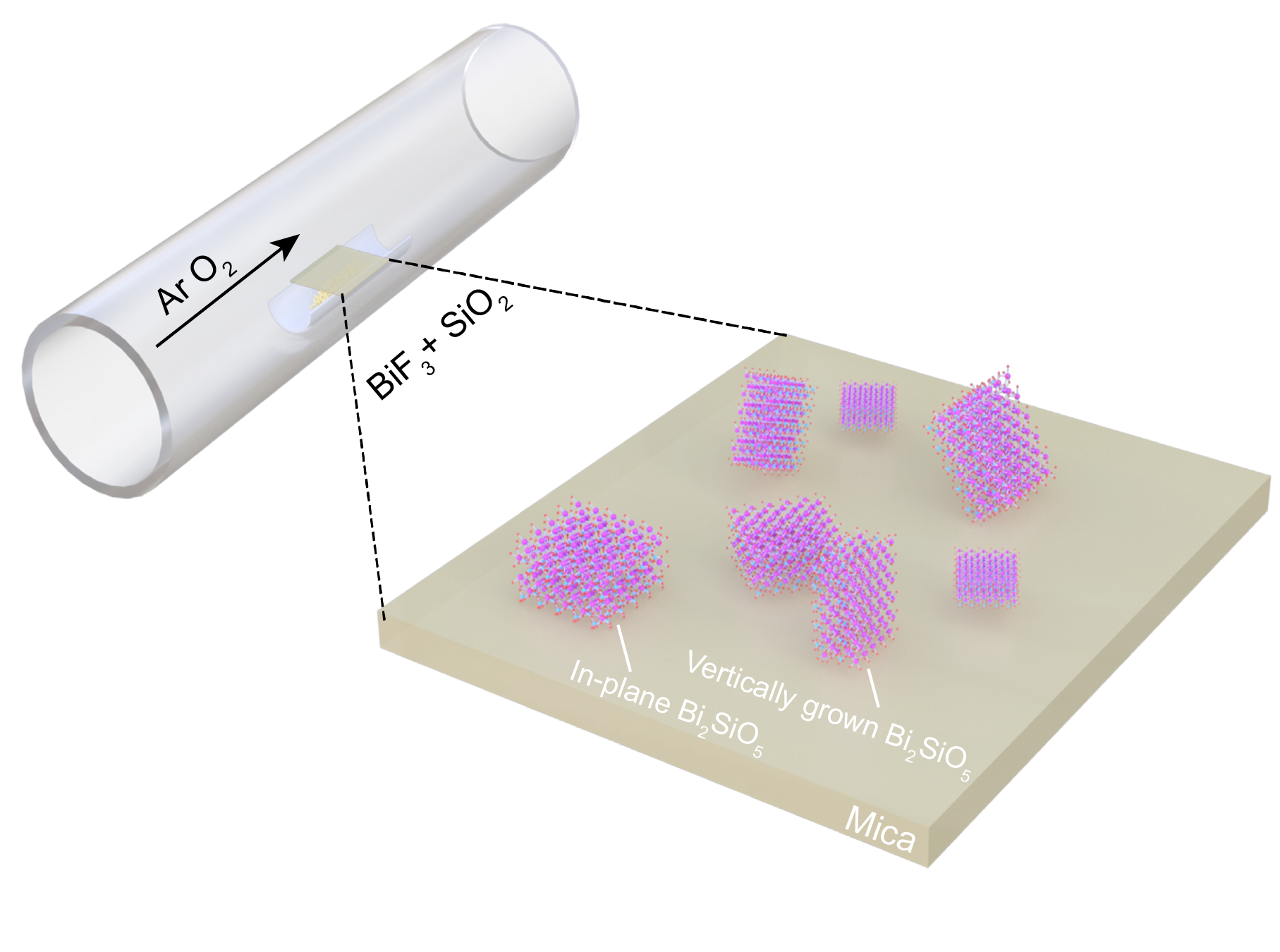


**Supplementary Fig. 1** | **Schematic illustration for the CVD growth of Bi_2_SiO_5_.** The BiF_3_ powders as Bi supplier and SiO_2_ powders or quartz boat as Si supplier were placed in a quartz boat located in the center of the heating area, and the freshly cleaved fluorophlogopite mica substrates were placed above quartz boat.


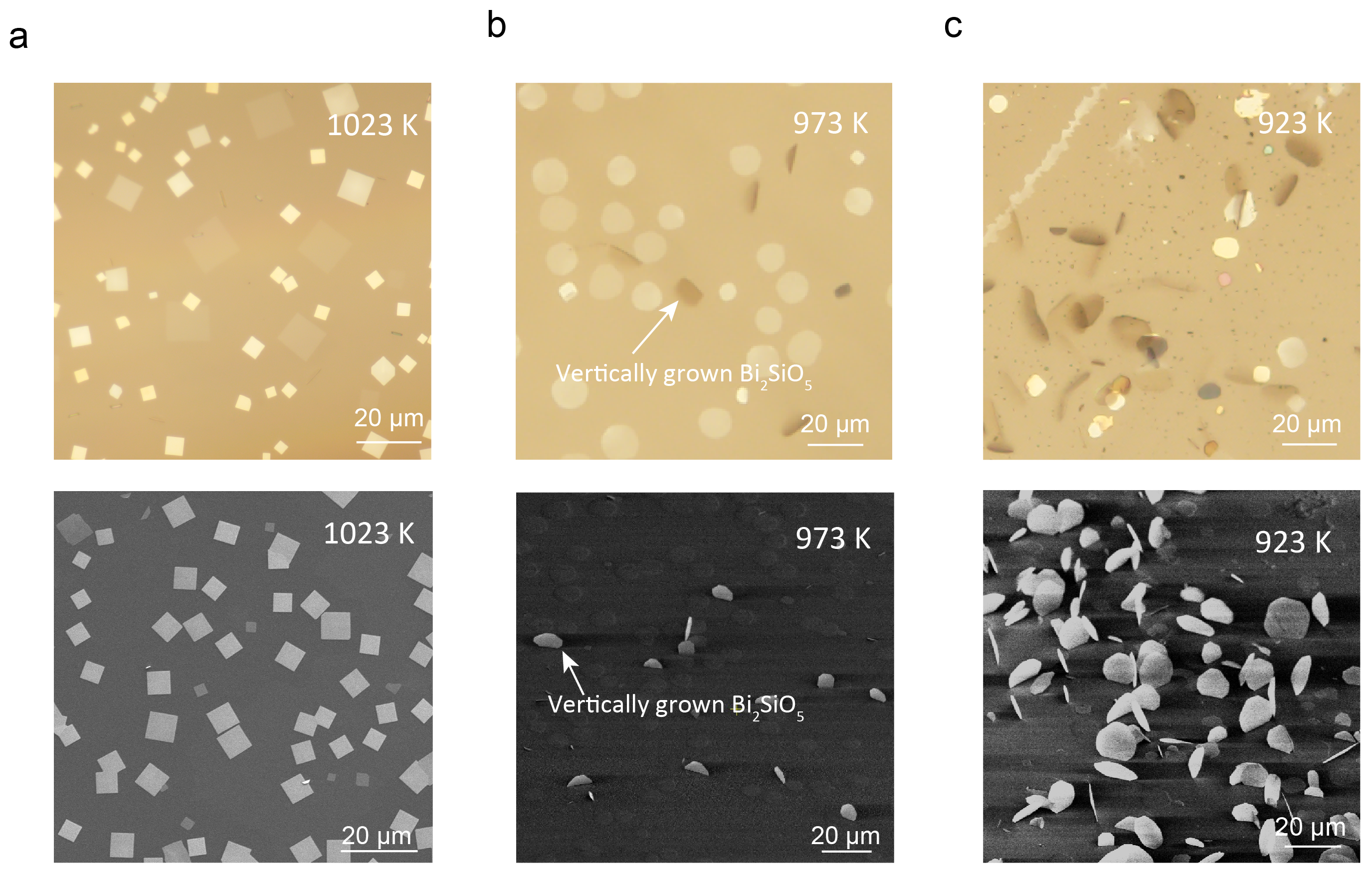


**Supplementary Fig. 2** | **Controlled synthesis of the in-plane and vertically grown Bi_2_SiO_5_ nanoflakes on mica with different temperatures.** By gradually increasing the growth temperature with other growth conditions fixed (such as pressure ~ 760 Torr, the flow rate of Ar ~ 50 sccm, trace amount of O2 (~100 ppm), locations of the source and the mica substrates and so on, different growth phenomena were observed in **a-c**. **a** In detail, at a higher temperature of 1023 K, the majority of Bi_2_SiO_5_ nanoplates followed the in-plane growth mode. **b** With the growth temperature decreasing to 973 K, the vertically grown samples emerge with the surrounding in-plane grown Bi_2_SiO_5_ nanoflakes . **c** With further decreasing the growth temperature to 923 K, more and more vertically grown Bi_2_SiO_5_ nanoplates appear and dominate the CVD growth. The top and bottom rows correspond to OM images and SEM images, respectively.


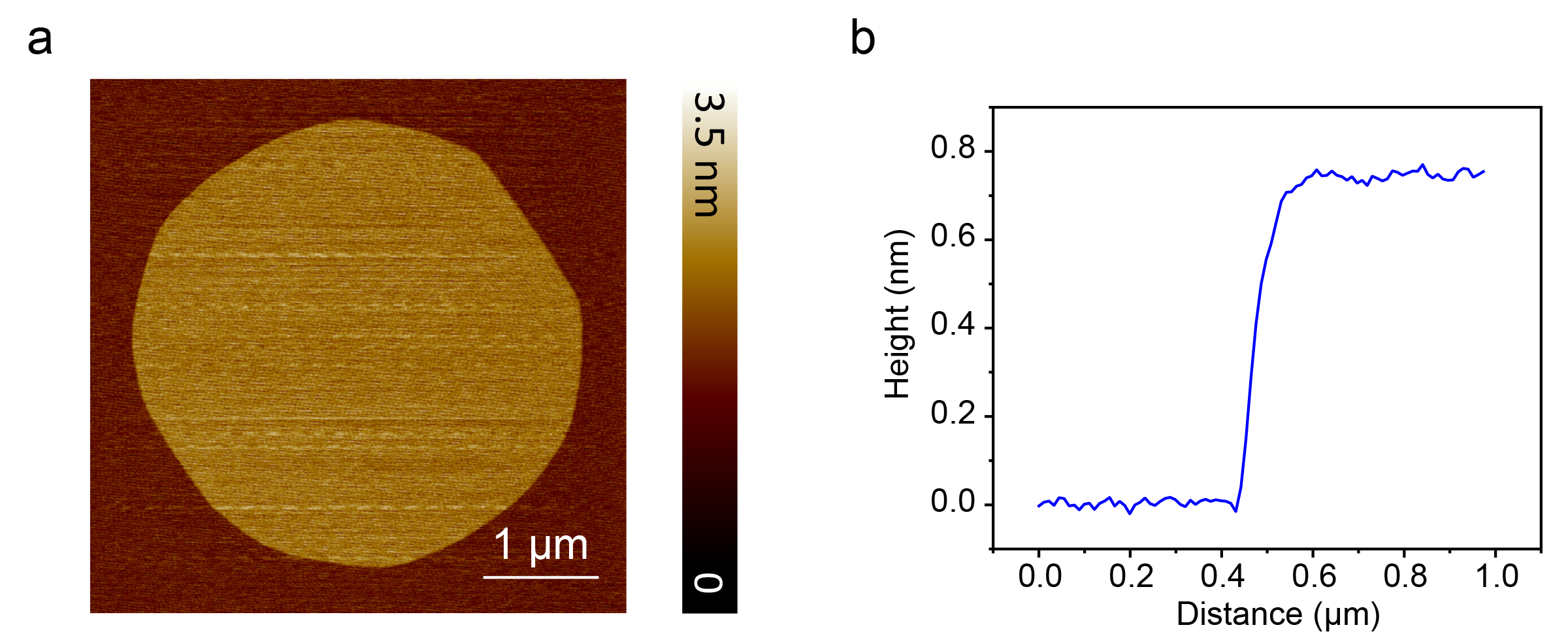


**Supplementary Fig. 3** | **AFM image and corresponding thickness profile of monolayer Bi_2_SiO_5_.**


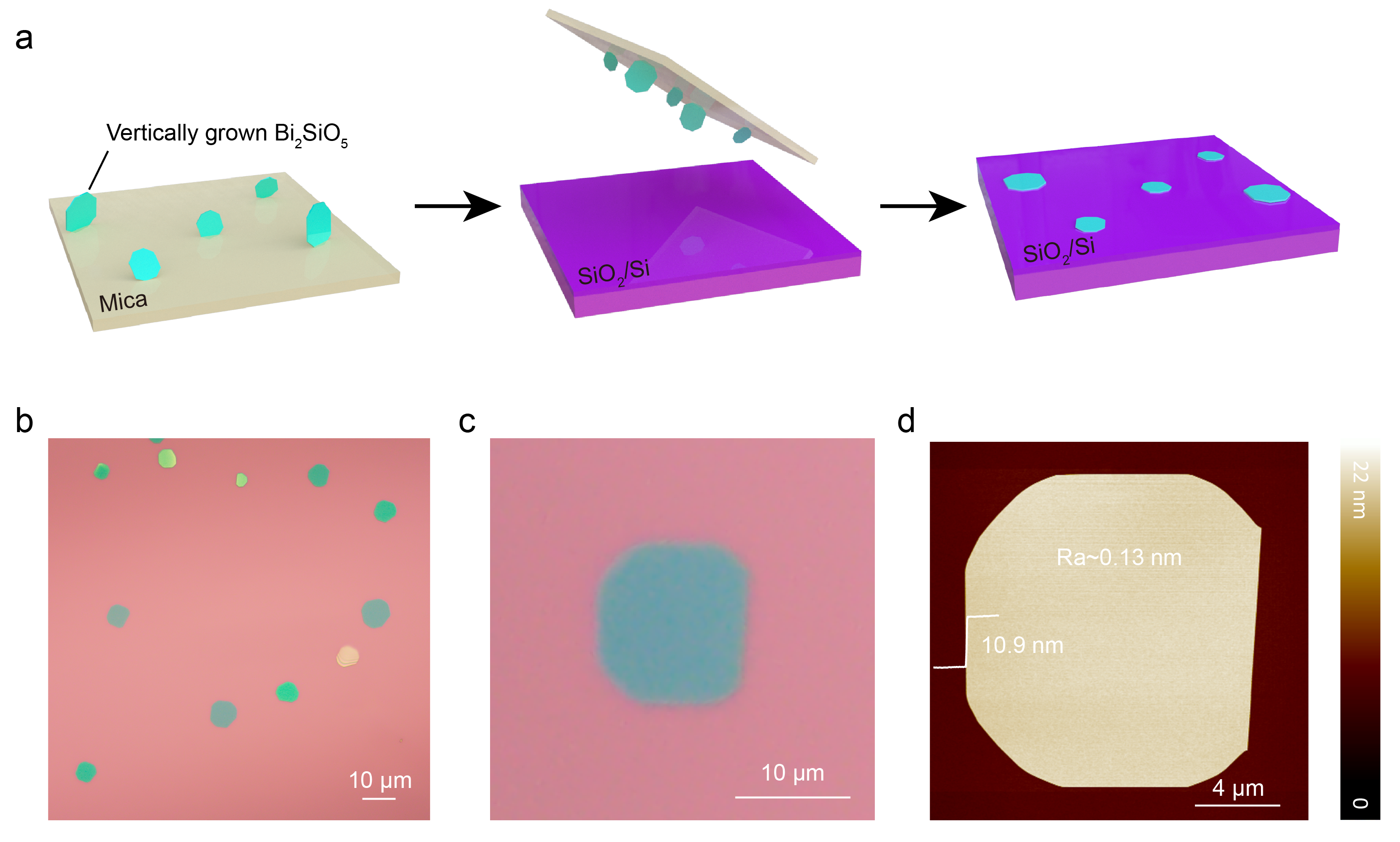


**Supplementary Fig. 4** | **Polymer-free transfer of vertically grown 2D Bi2SiO_5_ onto the SiO_2_/Si substrate by mechanical pressing.** **a** Cartoon of the mechanical pressing transfer method, by which the vertically grown Bi_2_SiO_5_ nanoplates on mica can be faithfully transferred onto other substrates, such as SiO_2_/Si. **b** A typical OM image of the Bi_2_SiO_5_ nanoplates transferred onto SiO_2_/Si. The color of different thickness Bi_2_SiO_5_ are apparent which can effectively help us to distinguish the thickness in subsequent device processing. **c, d** OM **c** and corresponding AFM **d** image of a

vertically grown Bi_2_SiO_5_ nanoplate with a thickness of 10.9 nm after transfer, preserving atomically smooth surface without polymer contaminations.


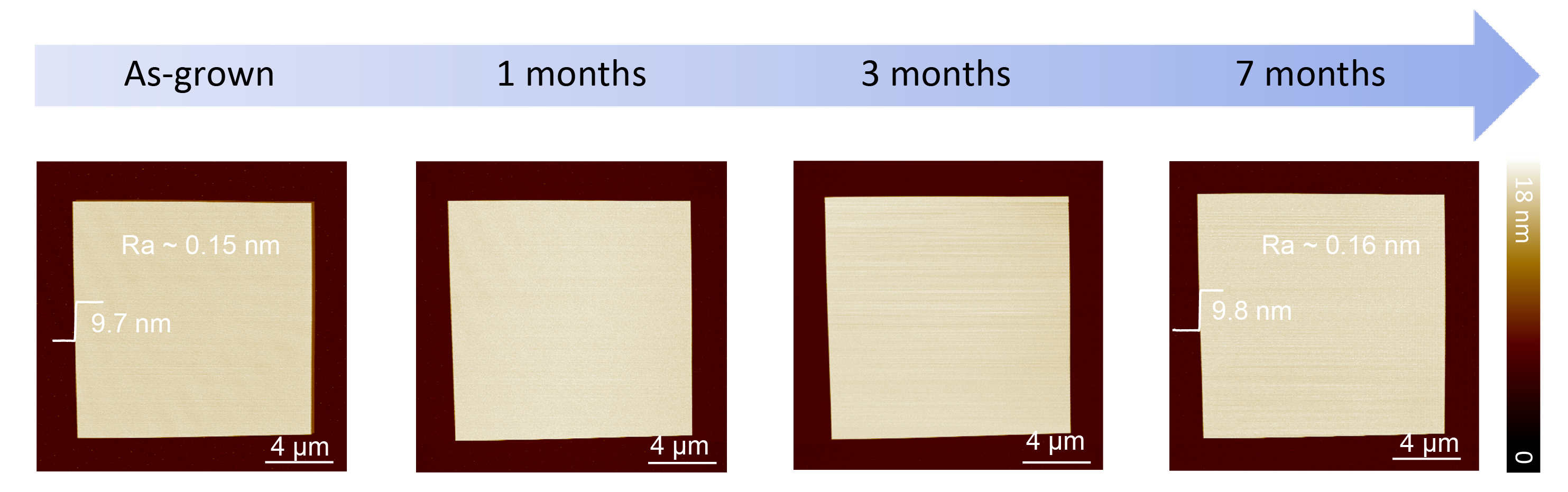


**Supplementary Fig. 5** | **The air stability of a 9.7-nm-thick Bi_2_SiO_5_ nanoplate on mica.** We monitored the surface morphology of a 9.7-nm-thick Bi_2_SiO_5_ nanoplate by AFM when exposed to air for more than 7 months. The surface always kept atomically smooth and had neglectable change after 7 months, suggesting the excellent environmental stability.


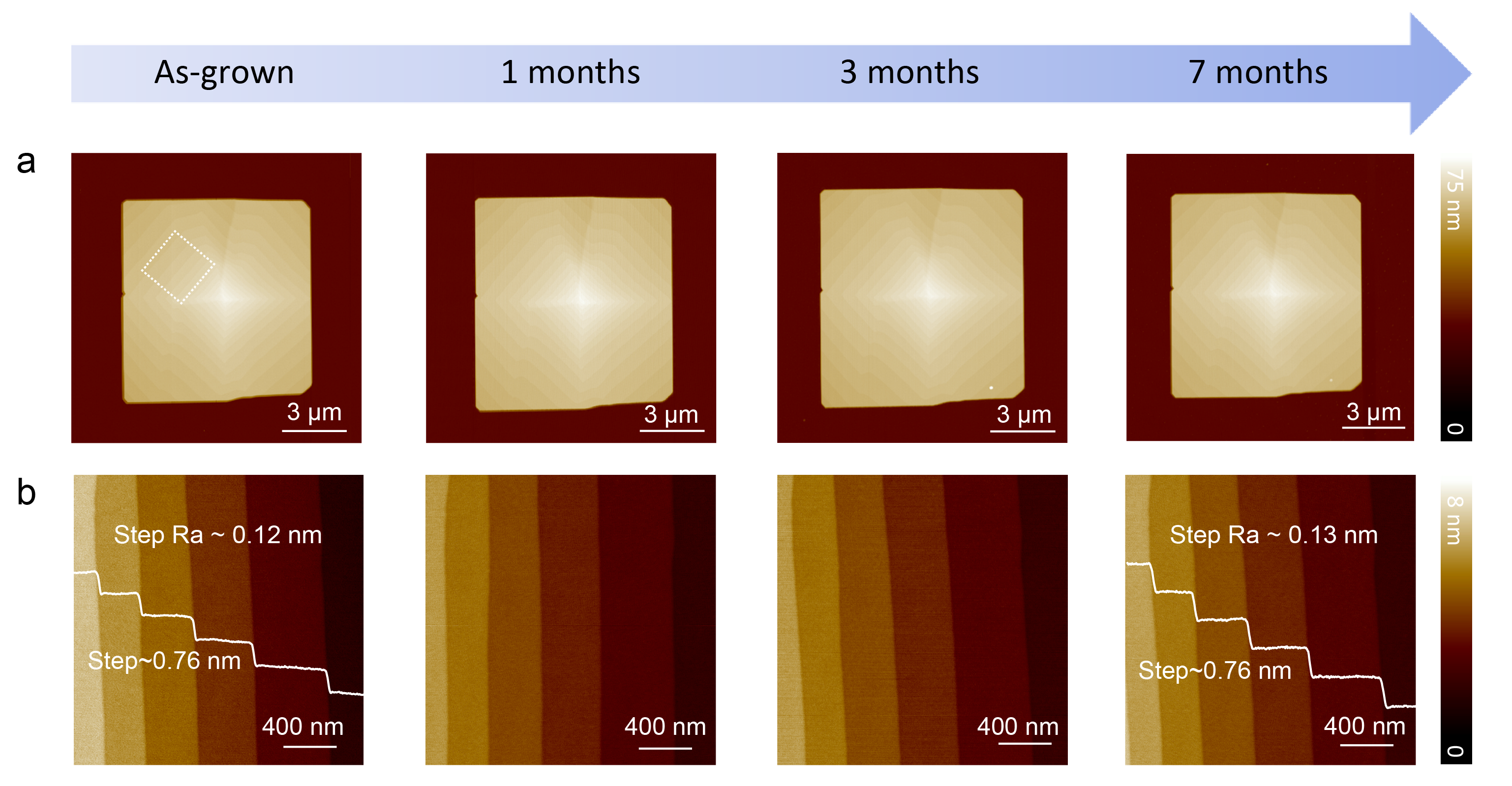


**Supplementary Fig. 6** | **The air stability of a CVD-grown terraced Bi_2_SiO_5_ nanoplate.** **a** AFM image of a CVD-grown Bi_2_SiO_5_ with clear terraces with different times of air exposure, such as 0, 1, 3 and 7 months. The thickness of this Bi_2_SiO_5_ nanoflake is 29.8 nm. **b** The corresponding AFM image of the enlarged terrace in **a**. Similar to the results in Supplementary Fig. 4, neglectable changes were observed both in the terrace roughness and height.

**Supplementary Note 2: Comparison experiments conducted to understand how the reaction goes in the CVD growth of Bi_2_SiO_5_ and the possible reason for its in-plane and out-of-plane growth.**

1. One step further to understand how the reaction goes in the CVD growth of Bi_2_SiO_5_?

First, it's well-known that the CVD growth usually undergoes a very complex microscopic process, including thermal decomposition/volatilization, gas transport, and absorption-diffusion-desorption of the chemical precursors in the form of atomic clusters. Even so, we can still try to understand the CVD growth process from the viewpoint that the compounds are formed by the combination of key elements-containing precursors. Undoubtedly, if one wants to synthesize the Bi_2_SiO_5_, it needs the Bi and Si precursors during the CVD growth process. Definitely, BiF_3_ powder is the only possible compound to supply the Bi-containing precursor. However, our CVD setup has a much more complex environment for Si. For example, the quartz boat, quartz tube and even the mica substrate can act as the Si-precursor supplier. To figure out the dominated Si-supplier, we performed extra comparison experiments. As shown in Supplementary Fig. 7, when the BiF_3_ powders were directly placed in the quartz boat container, Bi_2_SiO_5_ nanosheets can be readily obtained no matter on mica substrate (Supplementary Fig. 7a) or sapphire substrate (Supplementary Fig. 7b-c). If the quartz boat was replaced by the corundum boat (Al_2_O_3_) with SiO_2_ powders inside, we can still get the Bi_2_SiO_5_ phase (Supplementary Fig. 7d). However, if no SiO_2_ powders were placed in the corundum boat, we can't obtain the Bi_2_SiO_5_ phase at all, but a delta-Bi_2_O_3_ phase with a triangular like morphology was obtained instead (Supplementary Fig. 7e), whose crystal structure was verified by the cross sectional TEM characterizations (Supplementary Fig. 7f-h). The lattice spacings of 0.33 nm, 0.28 nm and 0.20 nm are consistent with (111), (002) and (220) planes in delta-Bi_2_O_3_. It suggests that the quartz boat may be the dominated parameter to supply Si and react with BiF_3_ to synthesize the Bi_2_SiO_5_ phase.


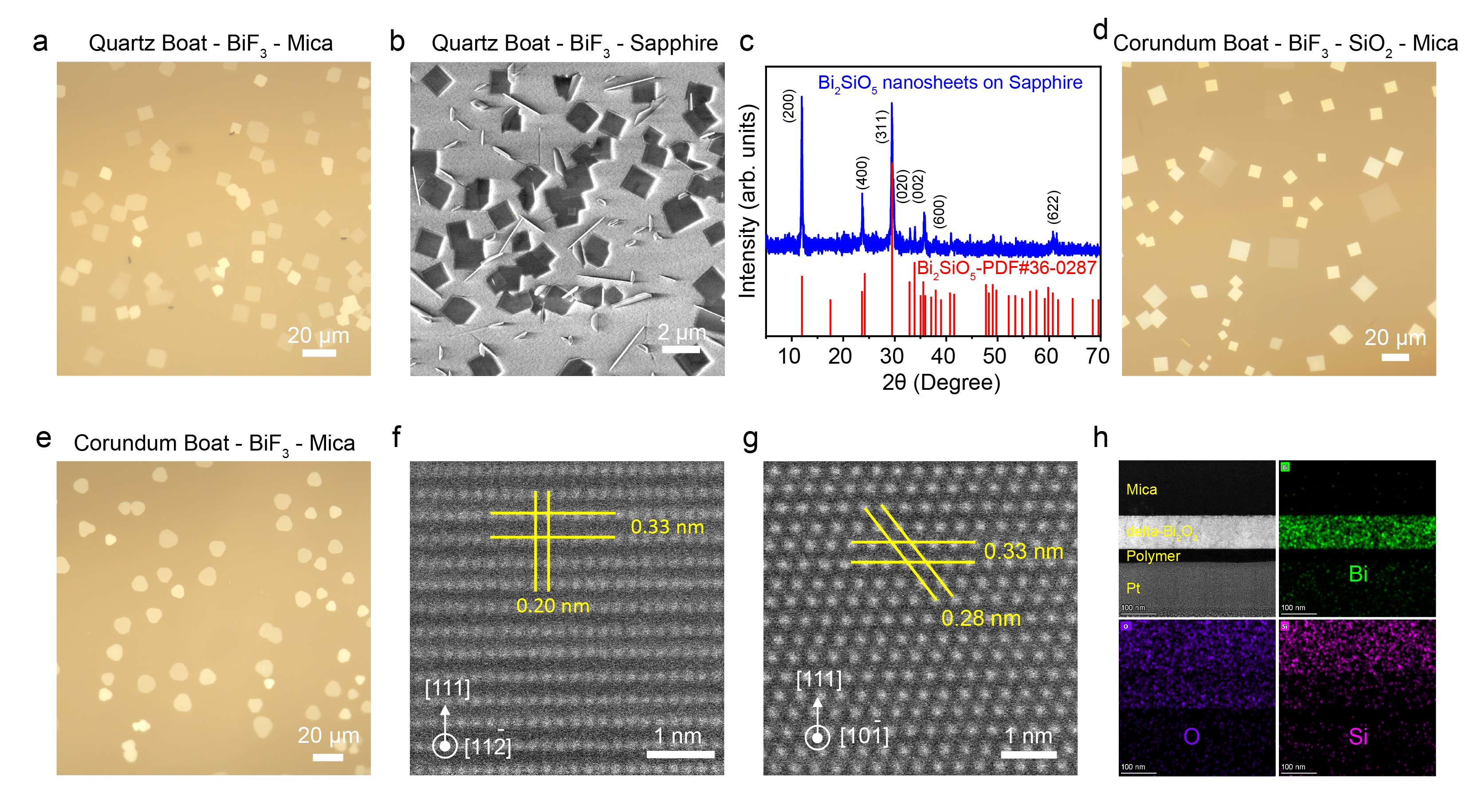


**Supplementary Fig. 7** | **Comparison experiments conducted to figure out which part of SiO_2_ dominates the CVD growth to synthesize the phase of Bi_2_SiO_5._** **a** A typical OM image of Bi_2_SiO_5_ nanosheets grown on mica substrate when the BiF_3_ powders are directly placed in the quartz boat container. **b** A typical SEM image of Bi_2_SiO_5_ nanosheets grown on sapphire substrate when the BiF_3_ powders are directly placed in the quartz boat container. **c** The corresponding XRD pattern of as-synthesized Bi_2_SiO_5_ on the sapphire substrate. **d** A typical OM image of Bi_2_SiO_5_ nanosheets grown on mica substrate when mixing the SiO_2_ powders with the BiF_3_ powders in the corundum boat. **e** A typical OM image of the delta-Bi_2_O_3_ phase with a triangular like morphology when the BiF_3_ powders are placed in the corundum boat container. **f, g** Cross-sectional TEM data of the triangular-like crystals imaging along the zone axes of $[11\bar{2}, \mathbf{f}]$, and $[10\bar{1}, \mathbf{g}]$, respectively. **h** The corresponding EDS elemental mappings for Bi, O and Si.

1. The possible reason for the in-plane and out-of-plane growth.

First, we should emphasize that we faithfully and repeatedly observe the phenomenon that lowering the growth temperature can regulate the CVD growth mode from in-plane to out-of-plane. As shown in Supplementary Fig. 8, we repeated each of CVD growth for in-plane and out-of-plane growth for 3 times, showing the very similar growth results. To this end, the question comes into why temperature matters for the growth mode of Bi_2_SiO_5_. Typically, the most direct way to address this issue is to calculate the nucleation energy barriers between different lattice planes of Bi_2_SiO_5_ and mica. However, this kind of calculation is quite challenging since we should know the exact form of the precursor clusters on mica or Bi_2_SiO_5_ surfaces during CVD growth. Here, we can do a qualitative analysis on this phenomenon. We should point out that altering the growth mode or aspect ratio of a nanomaterial by changing the synthetic temperature is a widely used method in other material systems. For example, Hong, C. Y. *et al*. reported the very similar temperature-induced vertical growth results in Bi_2_O_2_Se [*ACS Nano* 14, 16803-16812 (2020)], whose crystal structure is similar to Bi_2_SiO_5_. As we know, the species and relative partial pressure of the precursors would be different at different temperatures, which will also affect the nucleation energy barrier. Generally speaking, the vertical growth has a chemical bond-like interfacial interaction, which may lead to a lower nucleation energy barrier for vertical growth at low temperature. With growth temperature increasing, the adatoms (or precursor clusters) on the mica substrate are excited with higher kinetic energy to achieve a longer diffusion distance, and then the in-plane growth of Bi_2_SiO_5_ could be boosted.


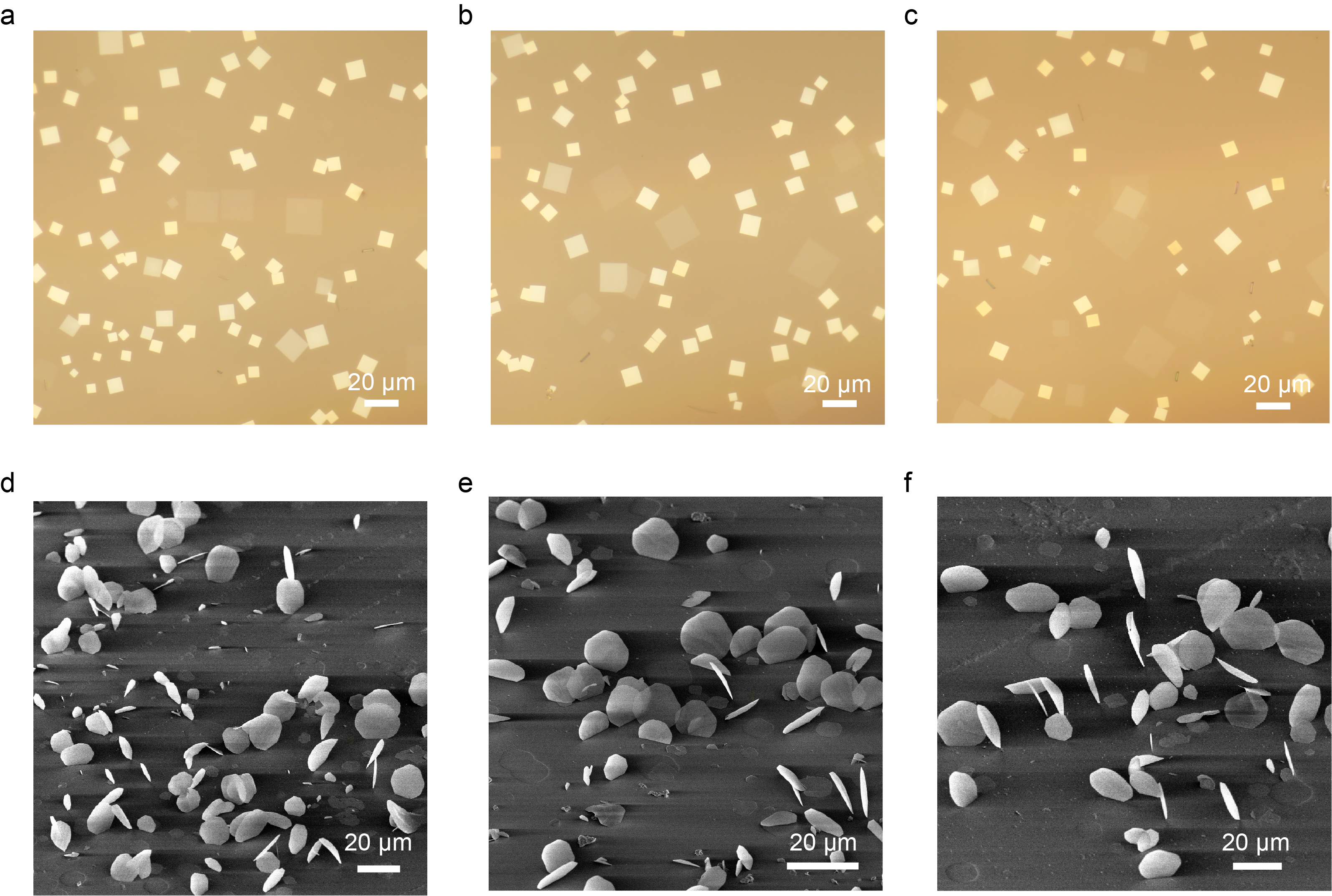


**Supplementary Fig. 8** | **Repeated CVD growth results of Bi_2_SiO_5_ with different growth modes.** Each of them was repeated for 3 times, showing very similar results. **a-c** OM images of in-plane grown Bi_2_SiO_5_ nanosheets on mica at a relatively high temperature of 1023 K. **d-f** SEM images of vertically grown Bi_2_SiO_5_ nanosheets on mica at a relatively low temperature of 923 K.

**Supplementary Note 3: Characterizations and electrical measurements on CVD-grown Bi_2_SiO_5_**

**
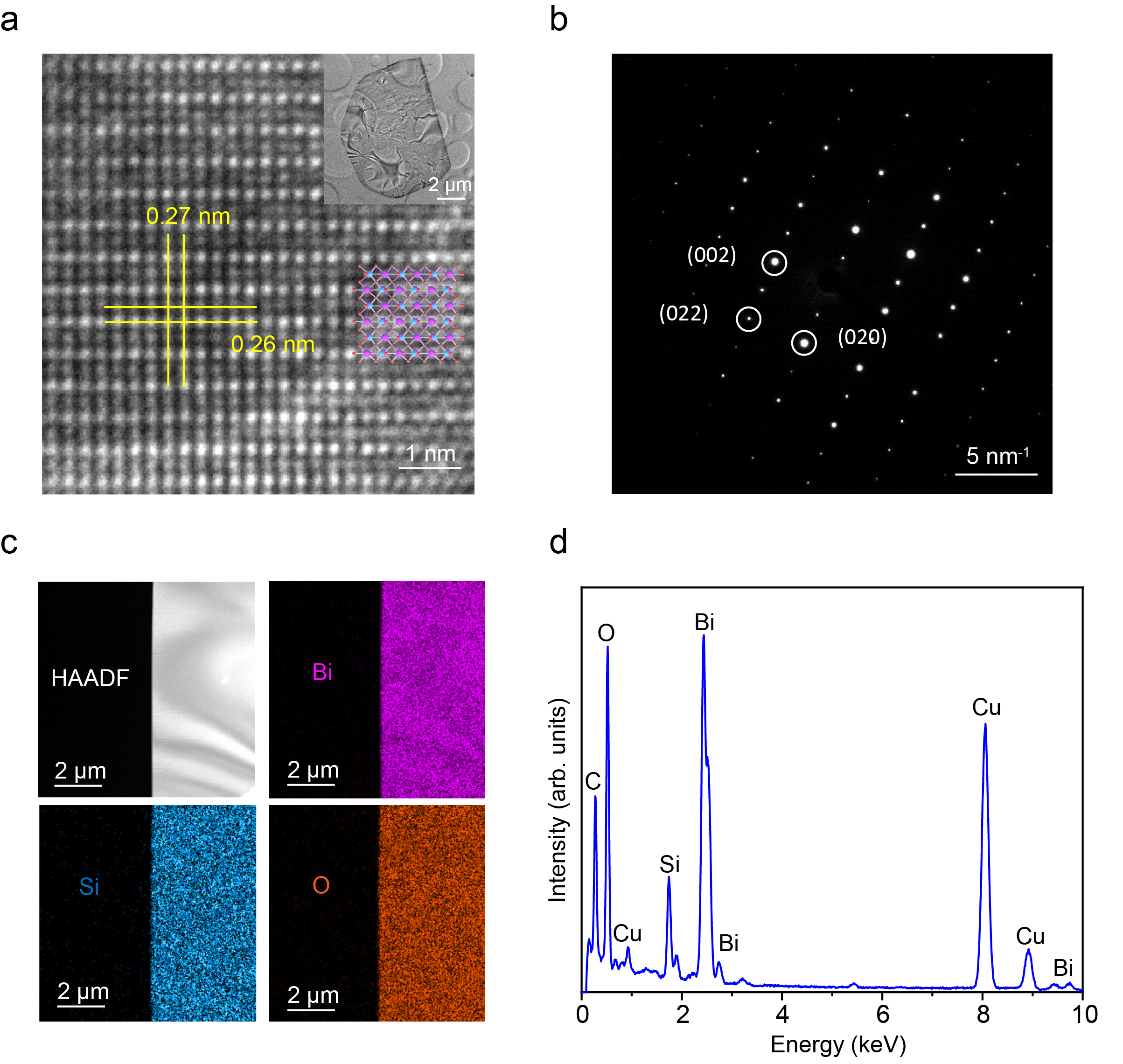
**

**Supplementary Fig. 9** | **TEM characterization of a Bi_2_SiO_5_ nanoplate transferred onto Cu grid.** **a, b** The bc-plane high-resolution TEM (HRTEM) image matches well with selected area electron diffraction (SEAD), whose lattice spacing of 0.26 nm and 0.27 nm ascribed to the (002) and (020) planes, respectively. The insert shows the low-magnification TEM image of the Bi_2_SiO_5_ nanoplate. **c** The high angle annular dark-field scanning TEM (HAADF-STEM) coupled with energy dispersive X-ray (EDX) elemental mapping revealed the uniform distribution of Bi, Si and O elements, with an averaged Bi/Si atomic ration of 2:1. **d** The corresponding EDX spectroscopy of the Bi_2_SiO_5_ nanoplate, in which the Cu signal comes from the Cu grid.

**
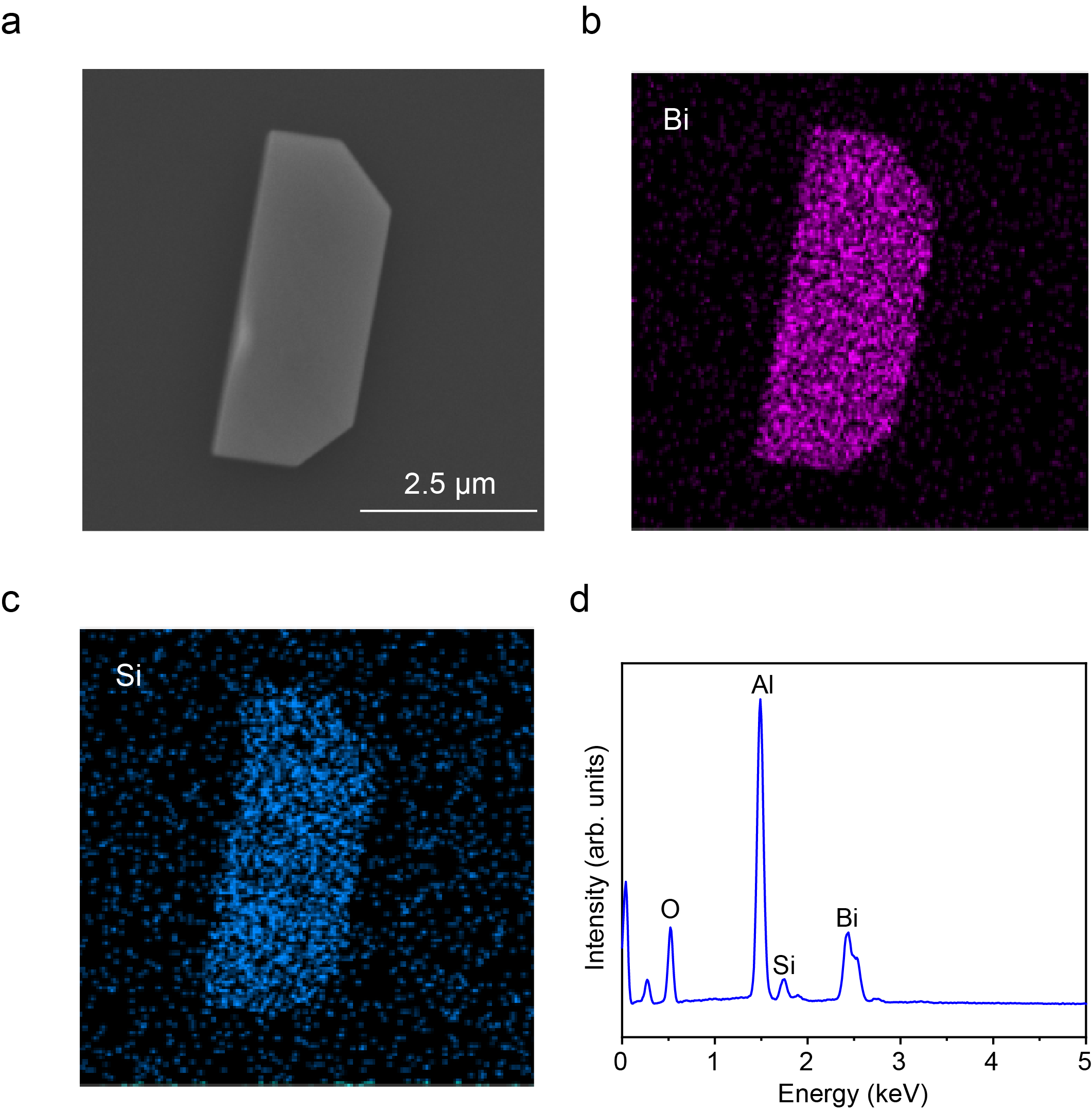
**

**Supplementary Fig. 10** | **SEM EDX mapping of a Bi_2_SiO_5_ nanoplate transferred onto a sapphire substrate.** **a-c** SEM image of a Bi_2_SiO_5_ nanoplate **a** and the corresponding elemental maps (**b, c**) for Bi, Si. **d** The corresponding EDX spectroscopy also gave an atomic ratio of ~2:1 for Bi: Si. The Al signal comes from the sapphire substrate.

**
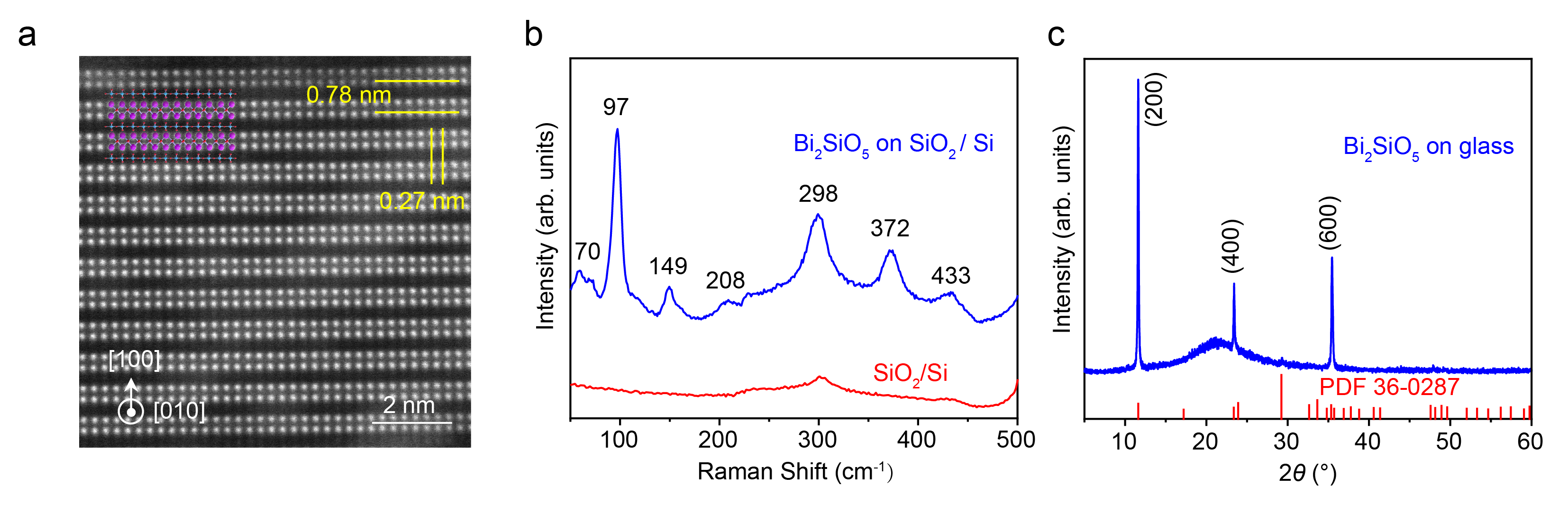
**

**Supplementary Fig. 11** | **Structural characterization of vertically grown Bi_2_SiO_5_ nanosheets by TEM, Raman and XRD. a** High-magnification cross-sectional HADDF-STEM image of a vertically grown Bi_2_SiO_5_ nanoflake, revealing the same layered structure as the in-plane grown Bi_2_SiO_5_. **b** Raman spectra of as-grown Bi_2_SiO_5_ nanoplate transferred onto the SiO_2_/Si substrate. The Raman spectra were measured by a 532 nm laser. The active modes at 70, 97, 149, 208, 298, 372, 433 cm^-1^ fit well with the monoclinic *Cc* phase of Bi_2_SiO_5_, which is a thermodynamically stable phase at room temperature. **c** The XRD pattern of the CVD-grown Bi_2_SiO_5_ nanoplates transferred onto glass substrate, showing strong (*h*00) peaks.

Particular attention should be paid on the equipment noise/offset level and detection limit when one wants to perform *C*-*V* measurements on a capacitor with ultrasmall capacitances. In fact, very strict and standard calibration processes were operated in all our *C-V* measurements. Specifically, we carefully measured the open-load capacitance to evaluate the total noise/offset level caused by the LCR meter, cables and probe stations. As shown in Supplementary Fig. 12a, our instrument has a capacitance offset of <1.5×10^-14^ F and capacitance noise of <0.9×10^-14^ F within the drive frequency from 100 Hz to 1 MHz. Comparatively, the absolute capacitance value of our Bi_2_SiO_5_-based MIM device is about 1.1×10^-12^ F, which is 2~3 orders higher than the equipment noise/offset level. Besides, the effective area (A) of MIM device (namely the overlap area between top and bottom electrodes) is estimated as 95 μm^2^. The thickness of the Bi_2_SiO_5_ is measured as 25.6 nm by AFM. Therefore, the dielectric constant is extracted as 32.4 based on the equation of $C =\frac{Aɛ_{o}ɛ_{eff}}{d}$.


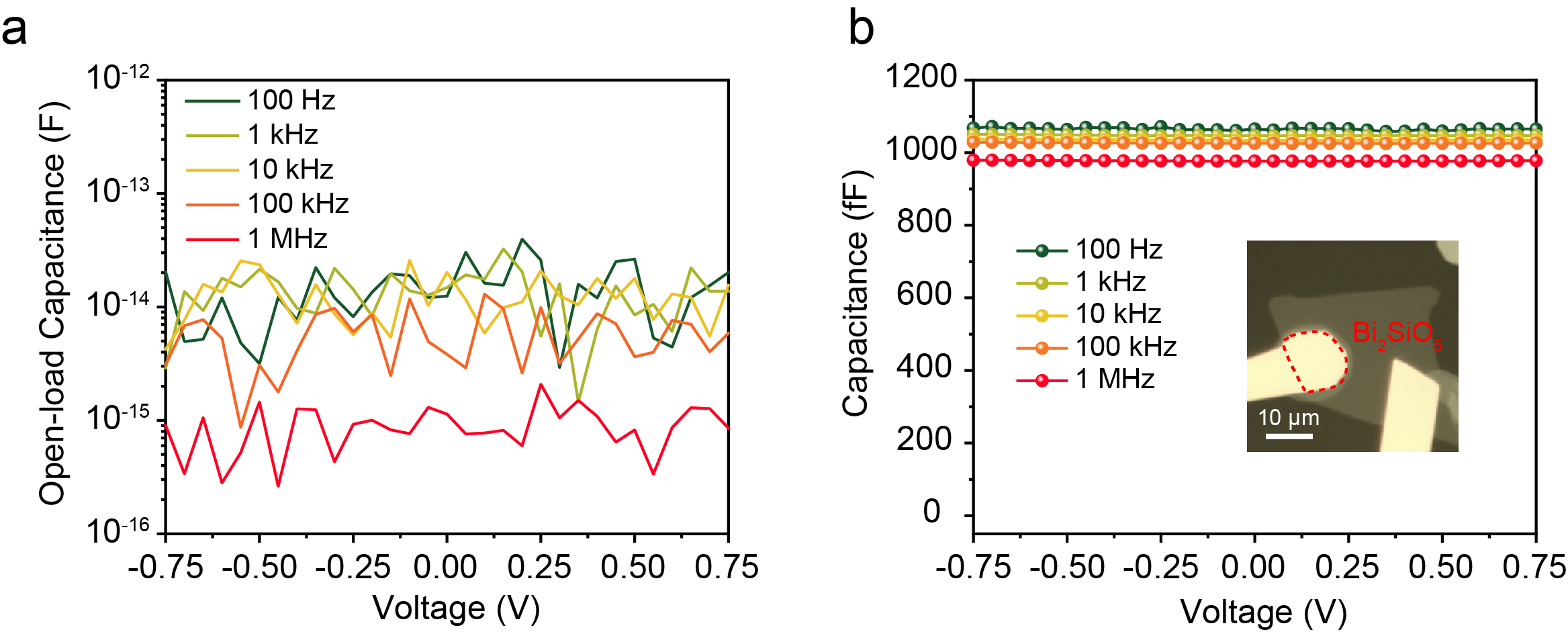


**Supplementary Fig. 12** | **a** The open-load *C*-*V* measurements within the drive frequency from 100 Hz to 1 MHz. **b** Raw data of the *C*-*V* measurements on a 25.6-nm-thick Bi_2_SiO_5_ nanoflake with a common MIM device configuration (Fig. 2a, main text), where the thick graphite and In/Au metals serve as the bottom and top electrodes (inset), respectively.


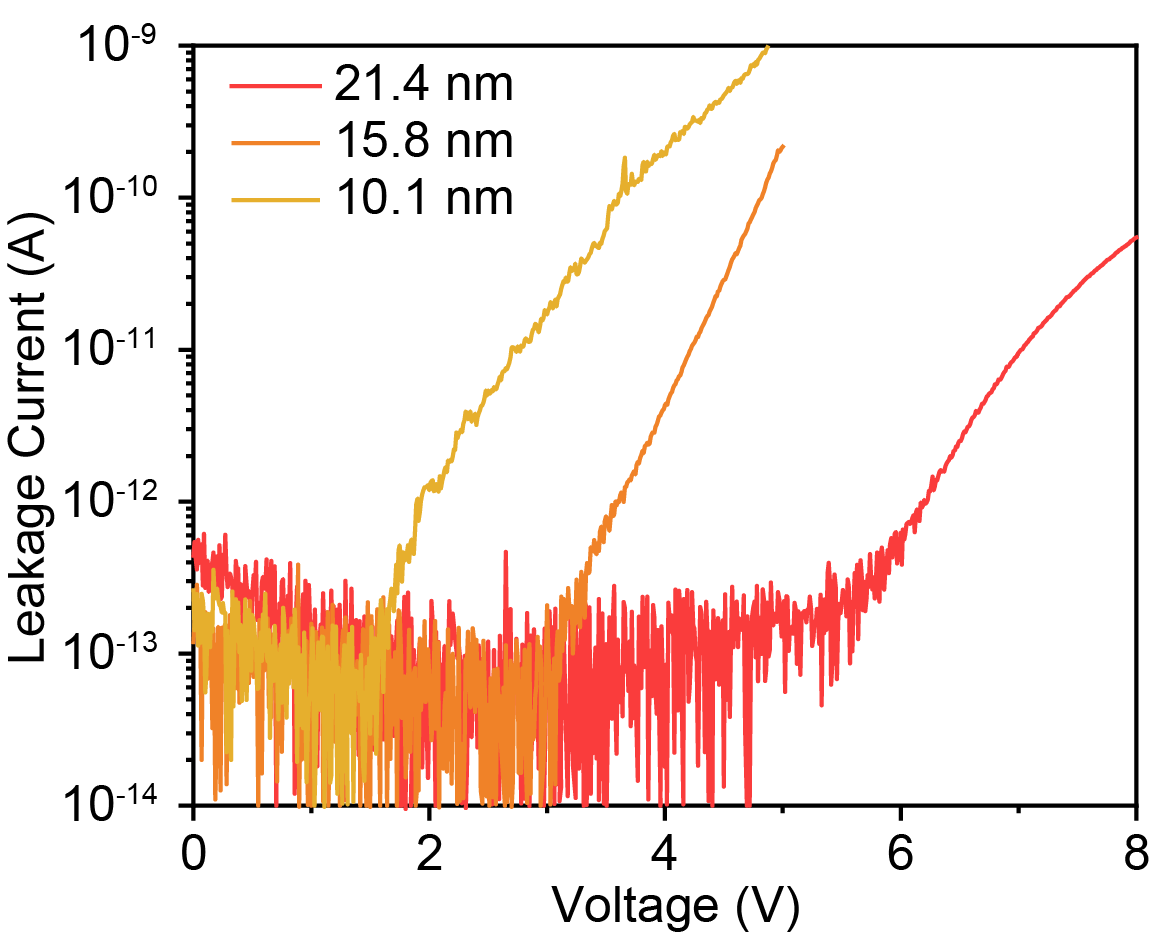


**Supplementary Fig. 13** | **The leakage current as a function of applied voltage for Bi_2_SiO_5_ nanoflakes with various thicknesses from 10.1 to 21.4 nm.**


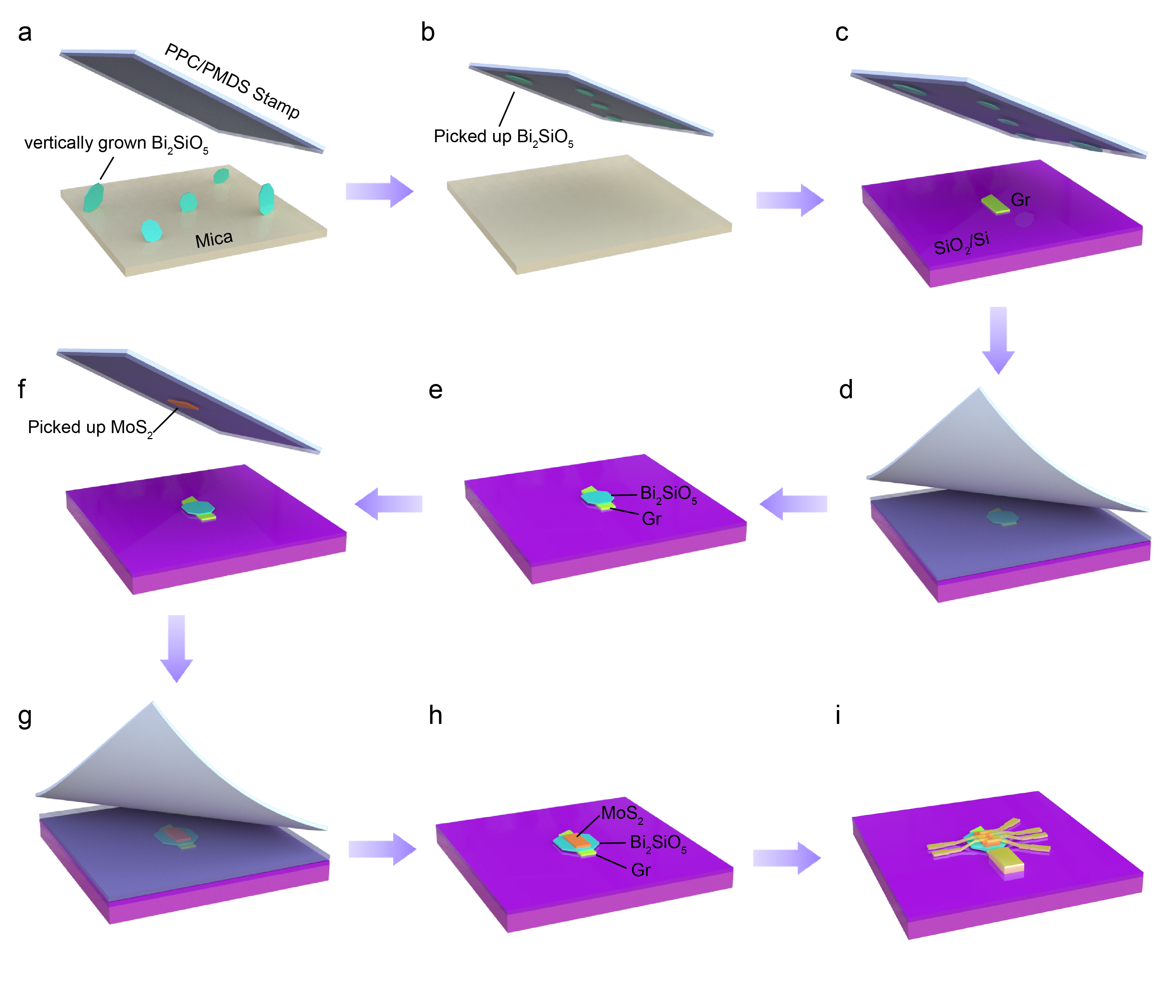


**Supplementary Fig. 14** | **The schematic illustration of fabricating MoS_2_ Hall-bar device with ultrathin Bi_2_SiO_5_ crystals as the high-*κ* back-gate dielectrics. a,b** First, we use the PPC/PMDS stamp to directly pick up the Bi_2_SiO_5_ nanoflake on the mica substrate. **c** The few-layer graphene was pre-exfoliated onto the well cleaned SiO_2_/Si substrate, followed by aligned transfer of Bi_2_SiO_5_ nanoflake onto the middle of the graphene nanosheet as the gate dielectric. **d** Then we peeled off the PDMS stamp by heating the substrate to 100 ℃ to reduce the vdw force between the PDMS and PPC, leaving the PPC/Bi_2_SiO_5_ on the SiO_2_/Si substrate. **e** Subsequently, the PPC was soaked in acetone for 5 minutes to clean up completely. **f-h** Next, with the similar process, the few-layer MoS_2_ was picked up by another PPC/PDMS stamp and transferred onto the middle of the Bi_2_SiO_5_ as the channel material. **g** Last, standard EBL process was used to pattern back-gate and Hall electrodes, followed by thermally evaporating In/Au (5 nm/40 nm) metals and lift off.


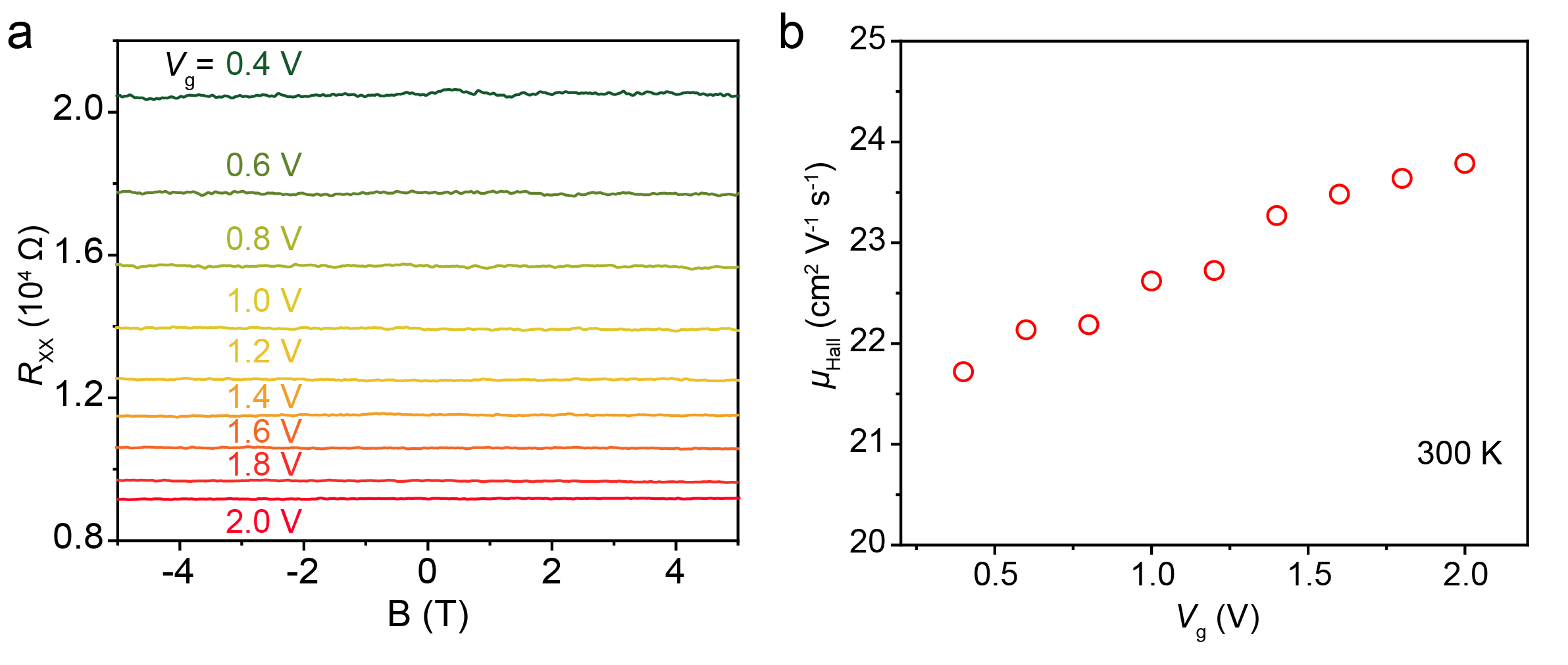


**Supplementary Fig. 15** | **Room-temperature gated Hall measurements on the Hall-bar device of Fig. 3 in the main text.** **a** *R*xx as a function of magnetic field at 300 K with different gate voltages. **b** Extracted Hall mobility as a function of gate voltage at 300 K. The Hall mobility gradually increased when a larger positive *V*_g_ was used, which can be ascribed to the carrier screening effect.

To check whether Bi_2_SiO_5_ can act as excellent gate dielectrics in short-channel FETs, we fabricate back-gate MoS_2_ FETs with short channel lengths, as demonstrated in Supplementary Figs. 16, 17. As we all know, fabricating short-channel device is quite technically challenging, which needs expensive lithography/evaporation facilities and well-trained operators. However, with great efforts, we have successfully scaled the channel length of MoS_2_ FET with graphene as the back-gate electrode to be 100 nm (Supplementary Fig. 16) and even 30 nm (Supplementary Fig. 17). Notably, we adopted the previously reported two-step EBL method [*Science.* 355, 271-276 (2017)] to separately deposit the source and drain electrodes, which is beneficial for the lift off of metal electrodes. As shown in Supplementary Fig. 16, the Bi_2_SiO_5_-gated MoS_2_ FET with a 100 nm channel length can be effectively switched on and off, exhibiting an on/off ratio of >10^8^, SS value of ~74 mV/decade, and DIBL value of ~30 mV/V. Furthermore, when the channel length is further scaled down to 30 nm, the Bi_2_SiO_5_-gated MoS_2_ FET still works well with a large on/off ratio. The relatively larger SS value of ~160 mV/decade and DIBL value of ~65 mV/V indicate substantial room space for device optimization. Besides, the On-state current of MoS_2_-based short channel length FET can be as high as ~35 μA/μm, which is believed to be further improved by precise contact engineering.


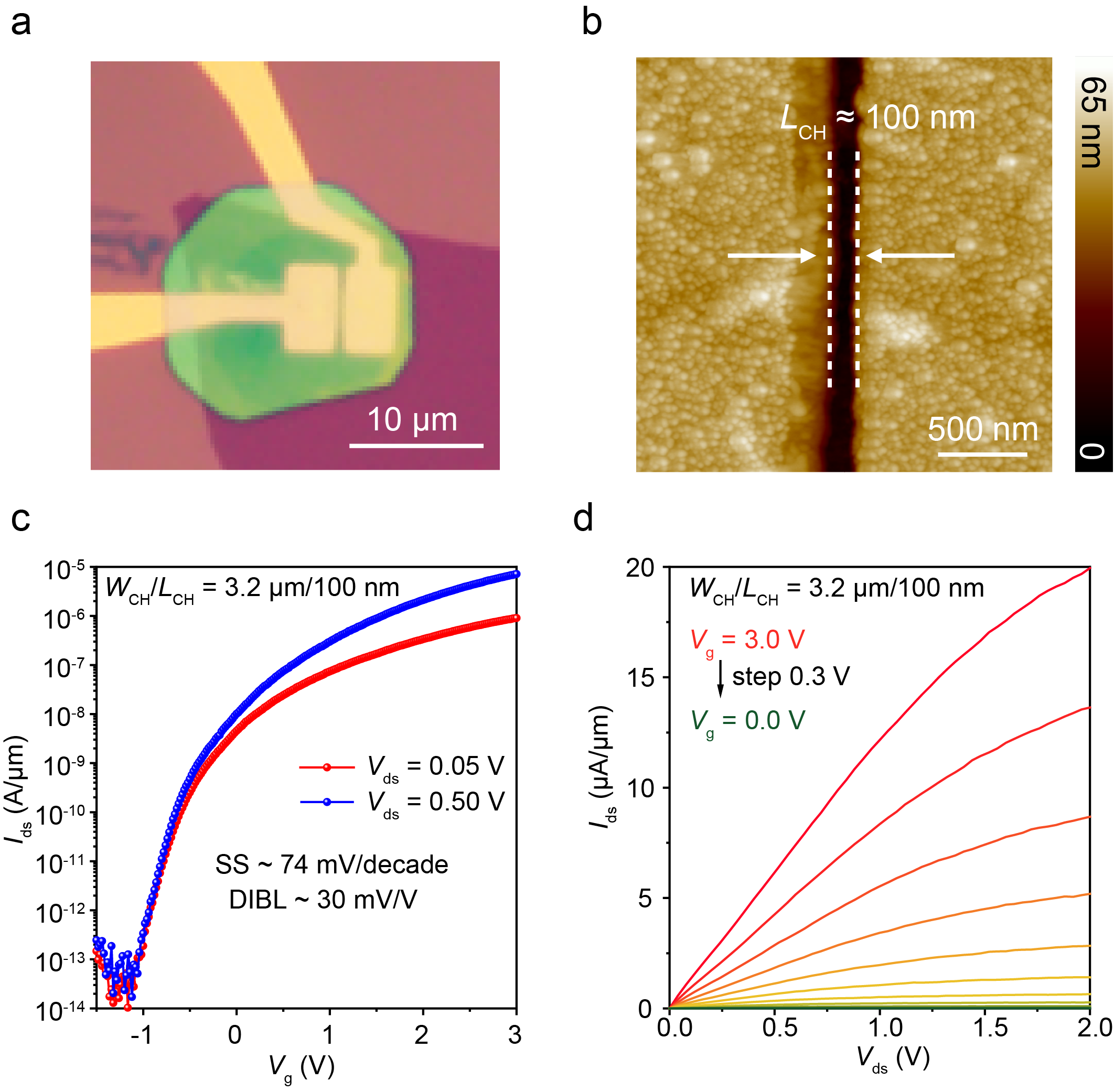


**Supplementary Fig. 16** | **A back-gate MoS_2_ short-channel FET (*L*_CH_ = 100 nm) with graphene and Bi_2_SiO_5_ as the back-gate electrode and dielectrics.** **a** OM image of the as-fabricated MoS_2_ FET with a channel length of 100 nm. **b** The corresponding AFM image, showing the channel length of ~ 100 nm. **c** Transfer characteristics of the device at *V*_ds_ = 0.05 V (red) and 0.50 V (blue), showing a large on/off ratio of >10^8^, small SS of 74 mV/decade and low DIBL value of 30 mV/V. **d** The corresponding output curves under different *V*_g_. From top to bottom, *V*_g_ varies from 3.0 to 0.0 V with a step of 0.3 V.


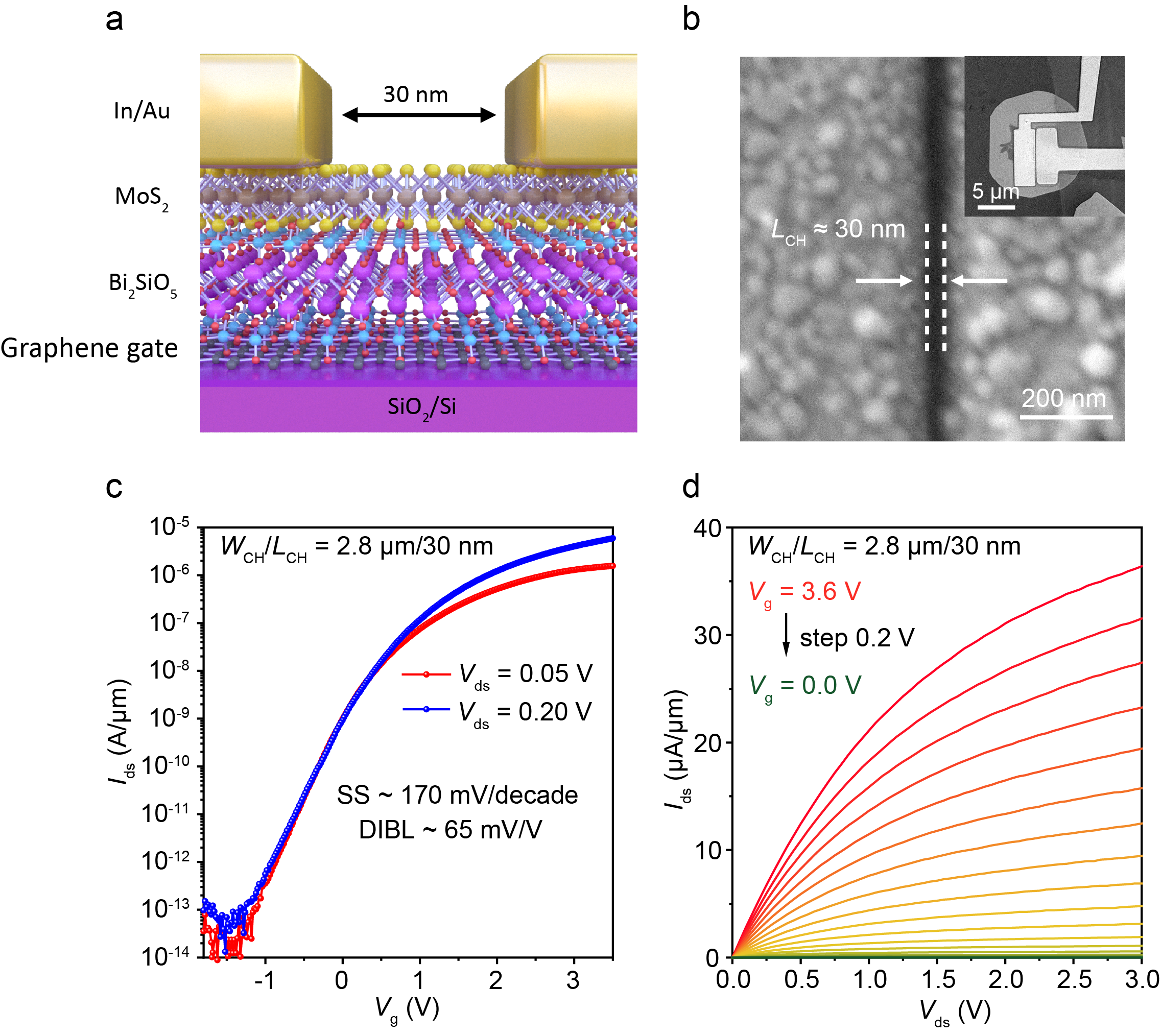


**Supplementary Fig. 17** | **Preliminary results of a back-gate MoS_2_ short-channel FET (*L*_CH_ = 30 nm) with graphene and Bi_2_SiO_5_ as the back-gate electrode and dielectrics.** **a** Schematic of the device structure. **b** SEM image of the as-fabricated MoS_2_ FET device, showing the channel length of ~30 nm. **c** Transfer characteristics of the device at *V*_ds_ = 0.05 V (red) and 0.20 V (blue), showing a large on/off ratio of >10^8^. Its SS value is ~170 mV/decade, and DIBL value is 65 mV/V. **d** The corresponding output curves under different *V*_g_. From top to bottom, *V*_g_ varies from 3.6 V to 0.0 V with a step of 0.2 V.


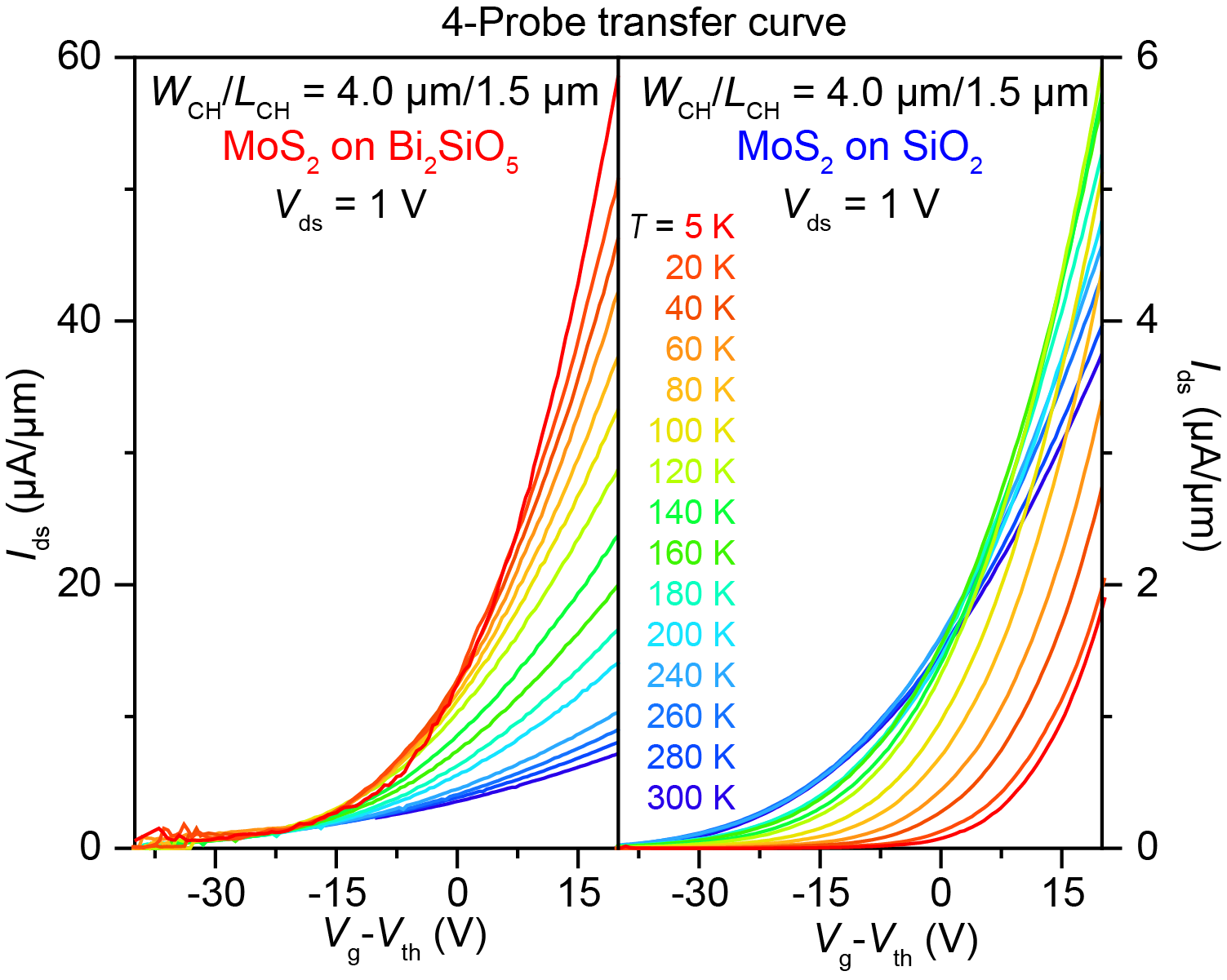


**Supplementary Fig. 18** | ***I*_ds_ as a function of *V*_g_-*V*_th_ in MoS_2_ FETs measured at different temperatures (5~300 K) on Bi_2_SiO_5_ (left) and SiO_2_ (right) substrates, respectively.**


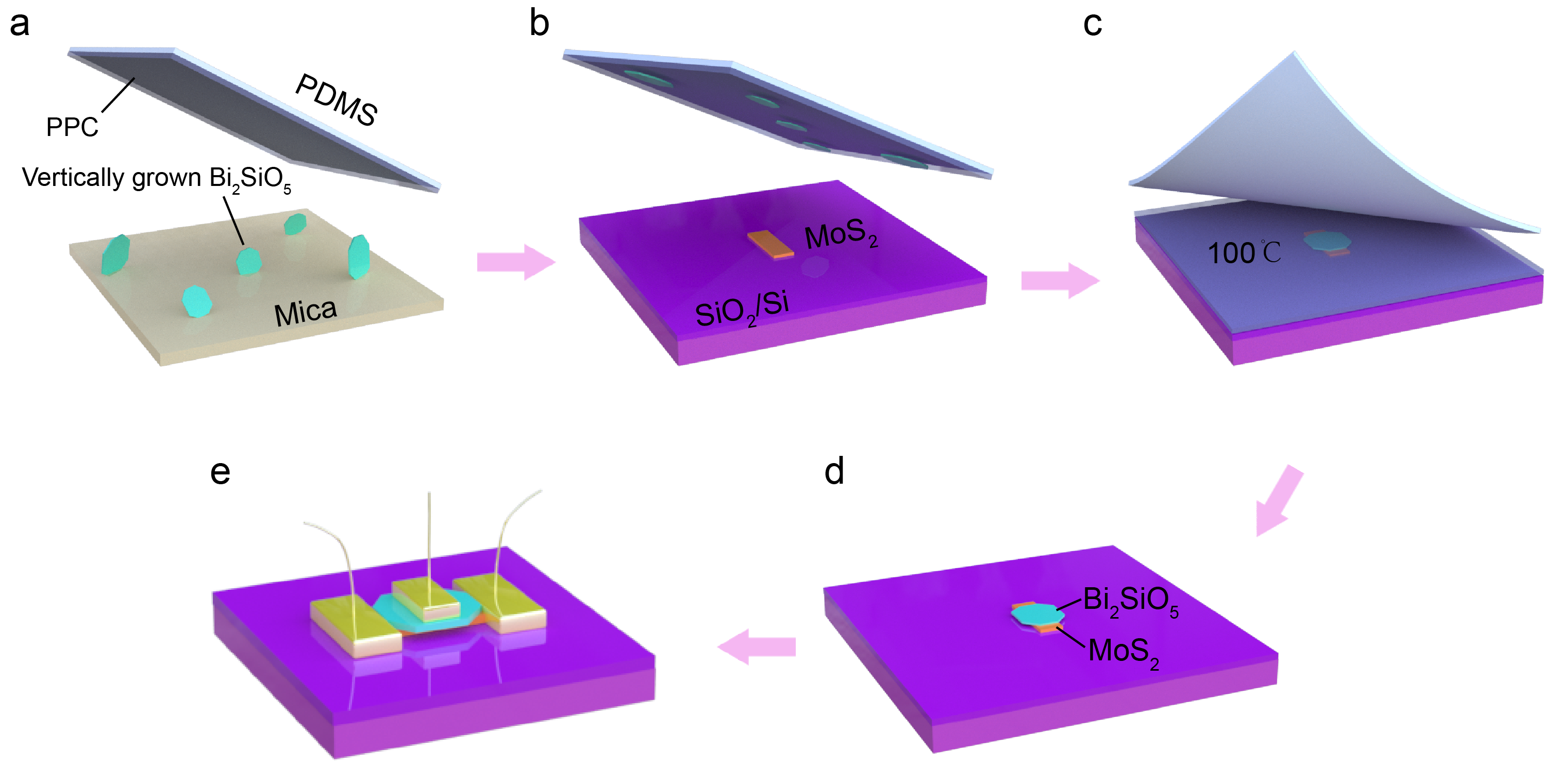


**Supplementary Fig. 19** | **The schematic illustration of fabricating the top-gated MoS_2_ FETs using Bi_2_SiO_5_ as the dielectric.** **a** First, we use the PPC/PMDS stamp to directly pick up the Bi_2_SiO_5_ nanoflake on the mica substrate. **b** The few-layer MoS_2_ were pre-exfoliated onto well cleaned SiO_2_/Si treated with O_2_ plasma for 5 min at a power of 50 W, followed by aligned transfer of Bi_2_SiO_5_ nanoflake onto the middle of the MoS_2_ nanosheet as the gate dielectric. **c** The PDMS stamp was peeled off by heating the substrate to 100 ℃ to reduce the vdw force between the PDMS and PPC, leaving the PPC/ Bi_2_SiO_5_ on top of the MoS_2_ naonsheet. **d** Subsequently, the PPC was soaked in acetone for 5 minutes to clean up completely. **e** Last, the source, drain and top gate electrodes were patterned together with one-step EBL process and thermal evaporation of In/Au metals (5 nm/40 nm).


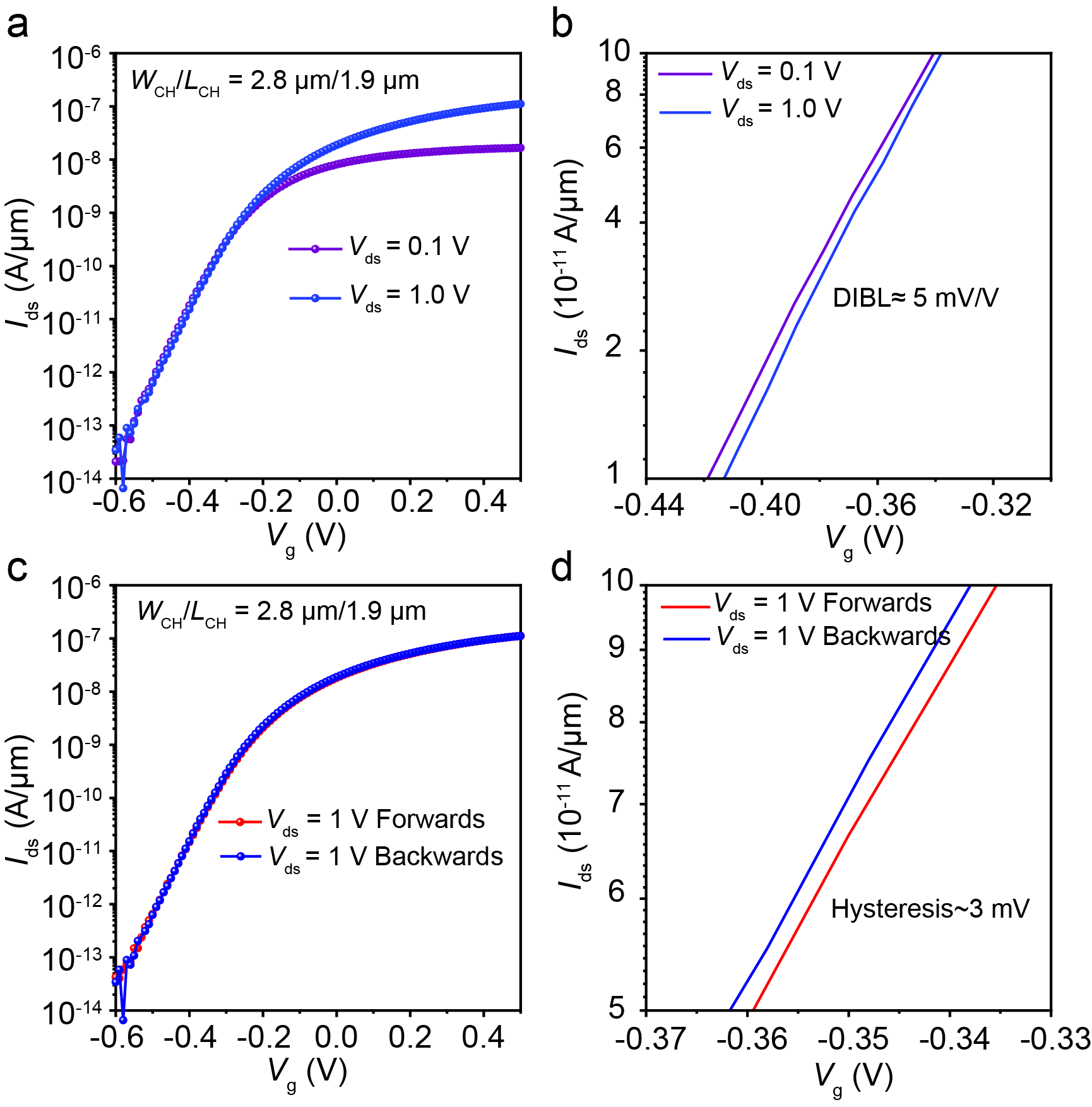


**Supplementary Fig. 20** | **Extraction of hysteresis and DIBL value from the transfer curves obtained in a top-gated MoS_2_/Bi_2_SiO_5_ FET (Fig. 5b, main text).** **a** The transfer characteristics operated at different *V*_ds_ to extract the DIBL value. *V*_ds_ = 0.1 V (purple) and 1.0 V (blue). **b** A low DIBL value of ≈5 mV/V was extracted from **a**. **c** Dual-sweep transfer characteristics obtained at *V*_ds_ =1.0 V of the same device in **a**. **d** The hysteresis between forwards and backwards sweeps is about 3 mV.


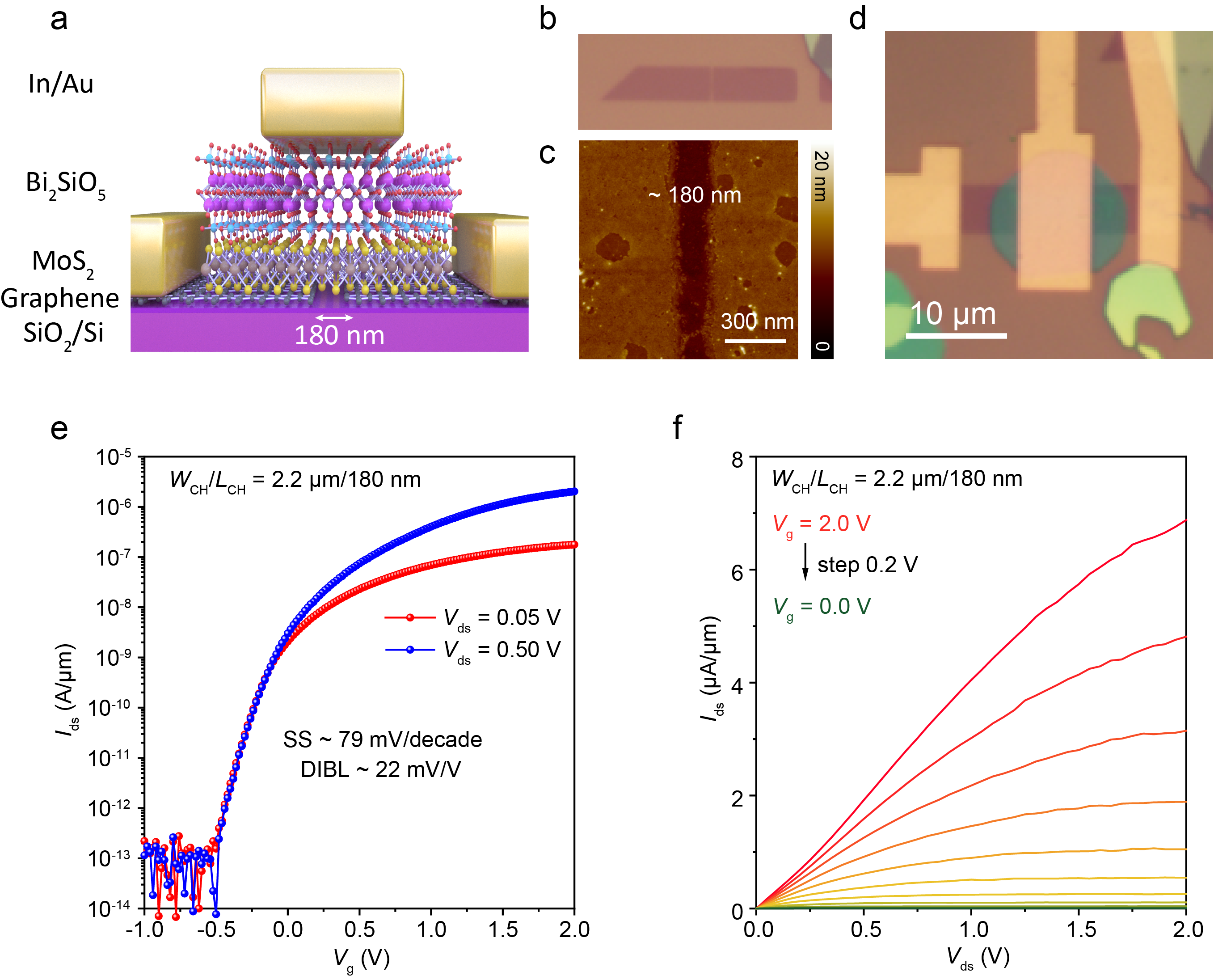


**Supplementary Fig. 21** | **A top-gate MoS_2_ short-channel FET with graphene as the contact electrodes and Bi_2_SiO_5_ as the gate dielectrics. a** Schematic of the device structure. **b, c** OM image (**b**) and AFM image (**c**) of the graphene electrodes with a gap distance of 180 nm, which was produced by EBL process and subsequent etching of O_2_ plasma. **d** OM image of as-fabricated Bi_2_SiO_5_-gated MoS_2_ short-channel FET with graphene as the contact electrodes. **e** Transfer curves of the device at *V*_ds_ = 0.05 V (red) and 0.50 V (blue), showing a small SS of 79 mV/decade and DIBL value of 22 mV/V. **f** The corresponding output curves under different *V*_g_. From top to bottom, *V*_g_ varies from 2.0 to 0.0 V with a step of 0.2 V.


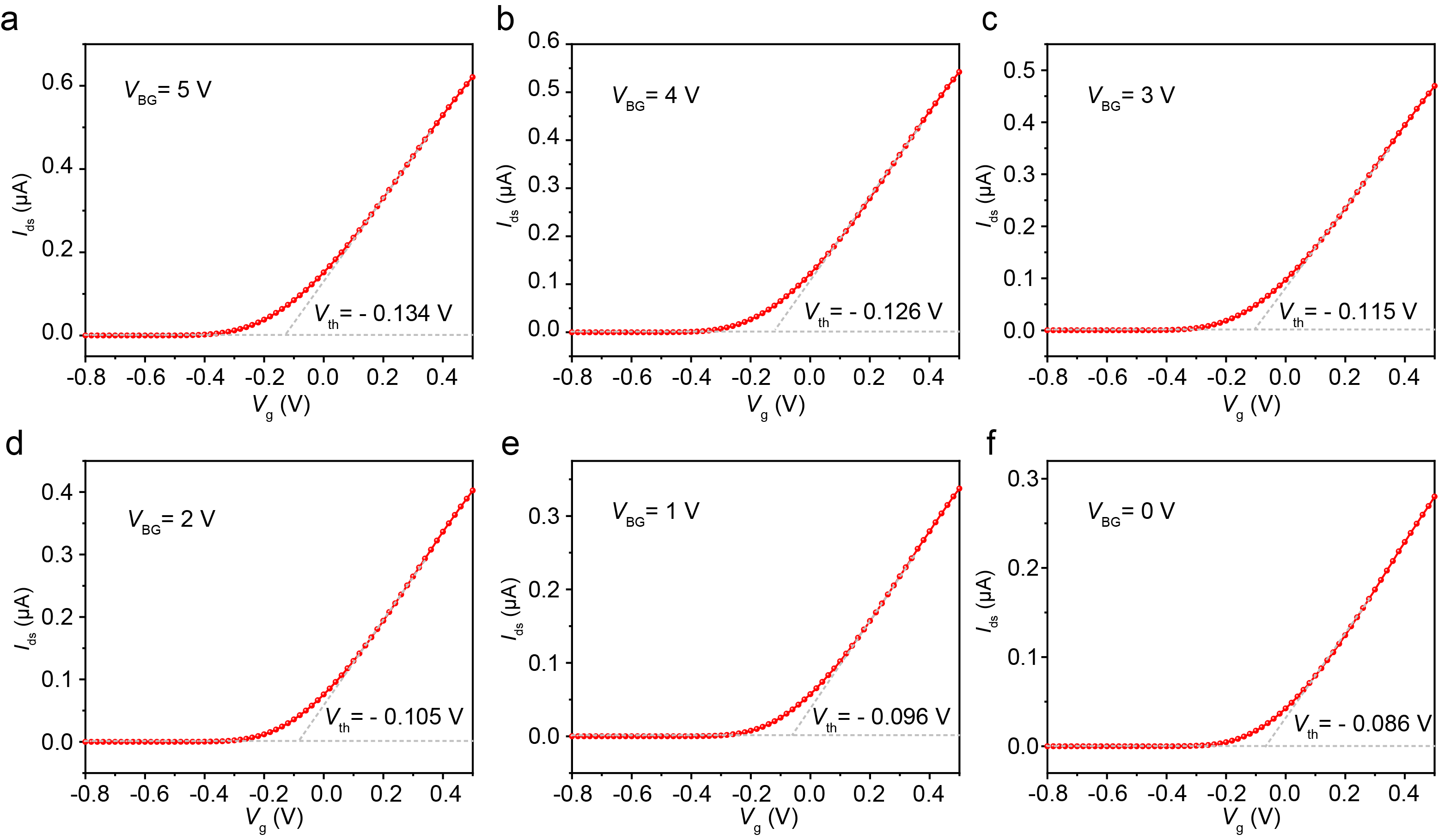


**Supplementary Fig. 22** | **The threshold voltage *V*_th_ exacted by linear fitting the transfer curves on a nonlogarithmic scale.** **a-f** Transfer curves of the device (raw data of Fig. 5e in the main text) under different back-gate voltages *V*_BG_ from 5 to 0 V.


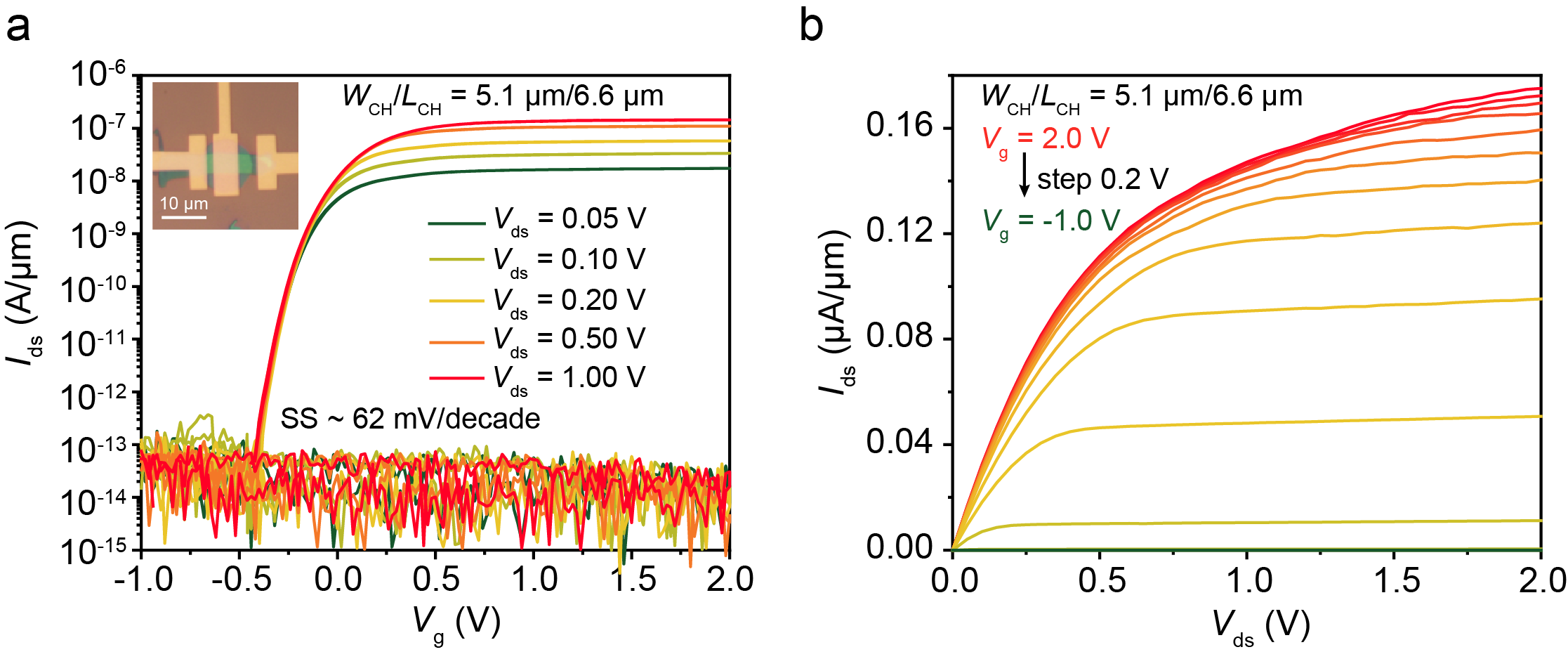


**Supplementary Fig. 23** | **Another top-gate MoS_2_ FET with 10-nm-thick Bi_2_SiO_5_ as gate dielectrics.** **a** Dual-sweep transfer curves of the MoS_2_/ Bi_2_SiO_5_ FET measured under different *V*_ds_ from 0.05 to 1 V, showing an ideal *SS* value of ~ 62 mV/decade and ignorable gate hysteresis. By pushing the gate voltage to 2 V, the *I*_ds_ saturated gradually. The OM image of the fabricated device is inserted in **a**. **b** Corresponding output curves of the device measured by varying the *V*_g_ from 2.0 to -1.0 V with a step of 0.2 V.


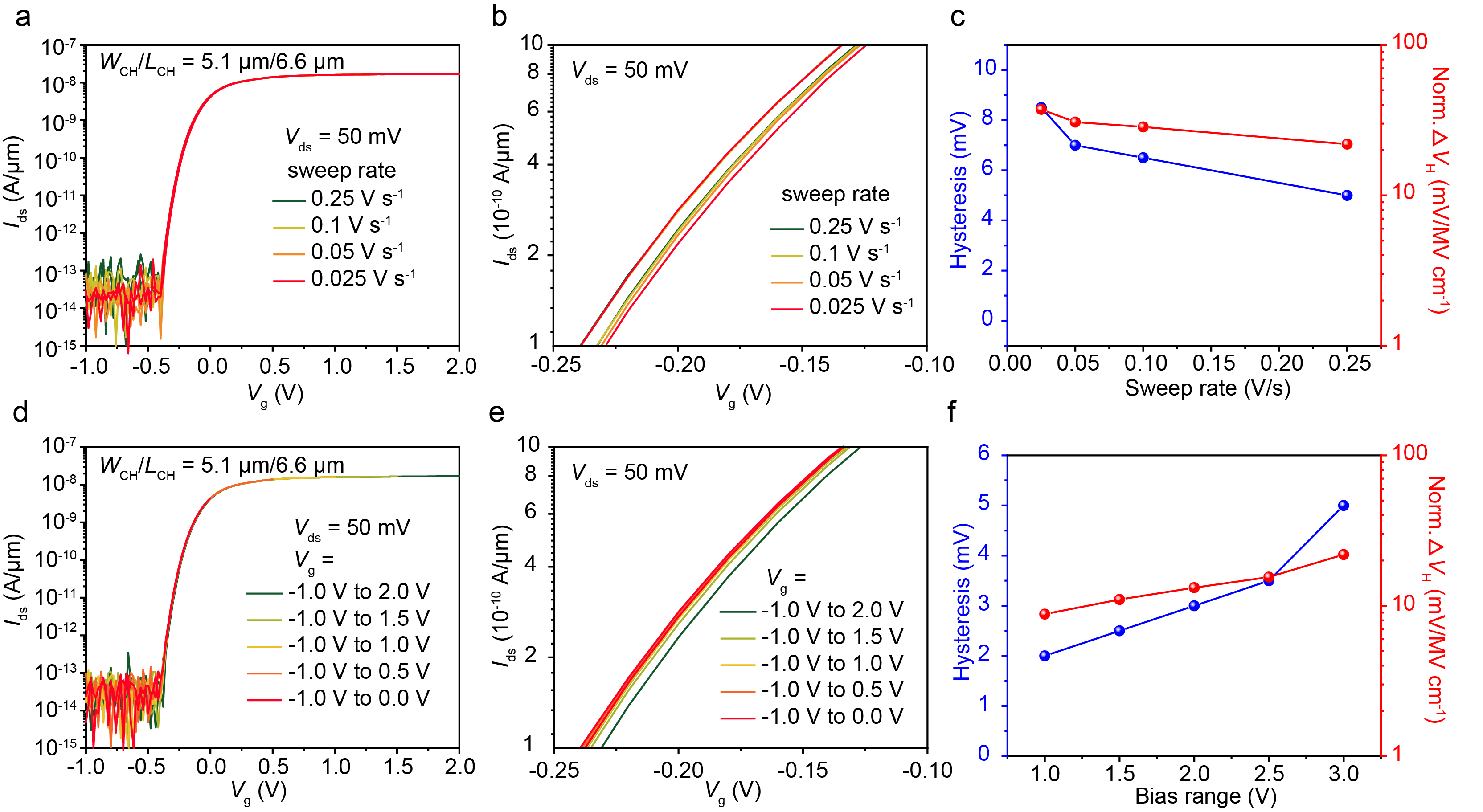


**Supplementary Fig. 24** | **a, b** Dual-sweep transfer characteristics of a top-gate MoS_2_ FET with the 10-nm-thick Bi_2_SiO_5_ under different sweep rates. The sweep speeds varied from 0.25 to 0.025 V s^-1^. **c** Extracted hysteresis and Normalized hysteresis at different sweep rates. **d, e** dual-sweep transfer curves measured under different *V*_g_ ranges. **f** Extracted hysteresis and Normalized hysteresis at different *V*_g_ ranges.


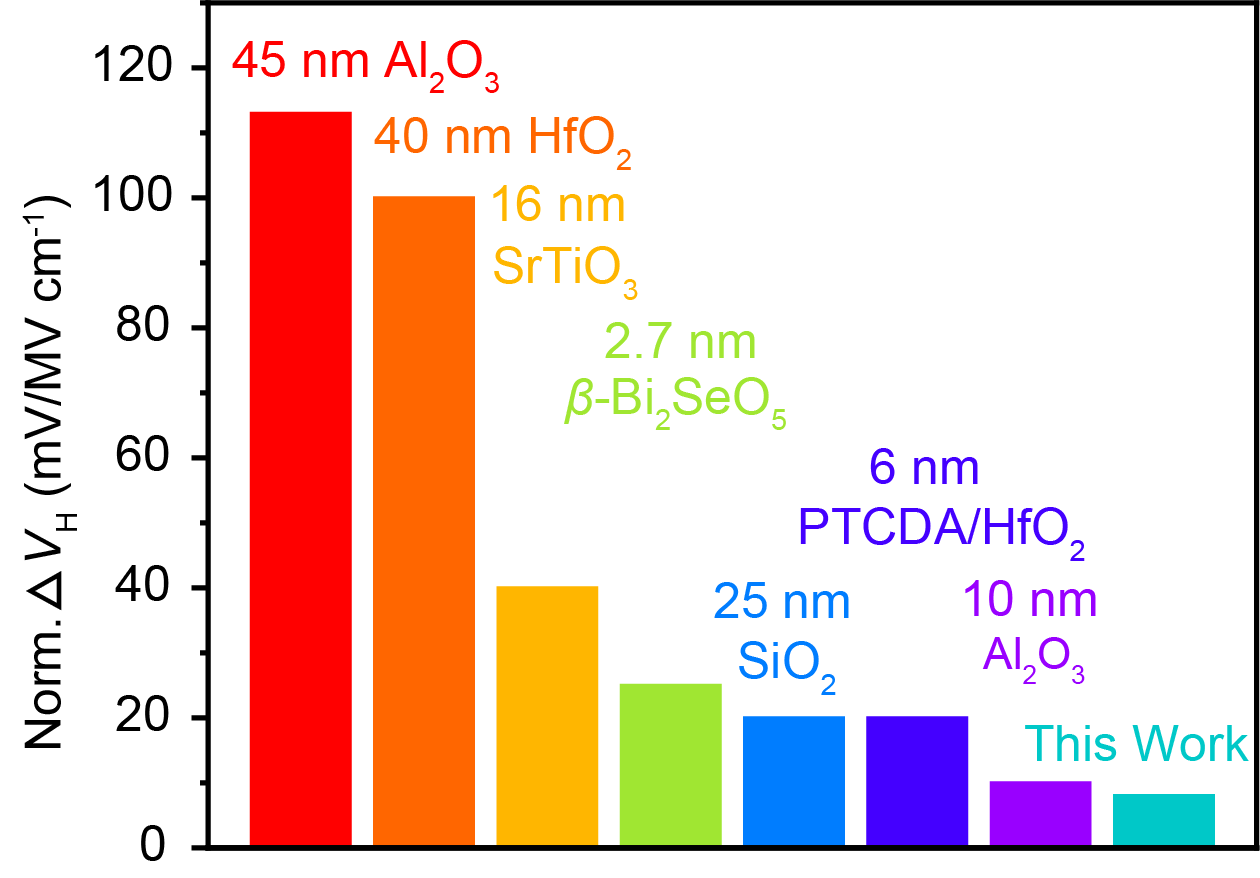


**Supplementary Fig. 25** | **Comparison of normalized hysteresis in our device with other literatures** [*Nat. Commun.* 14, 2340 (2023); *Nature* 605, 262 (2022); *Nat. Electron.* 5, 643 (2022); *Nat. Electron.* 2, 563 (2019); *AIP Adv.* 5, 057102 (2015); *IEEE Electr. Device L.* 38, 1763 (2017); *Appl. Phys. Express* 9, 095202 (2016)].


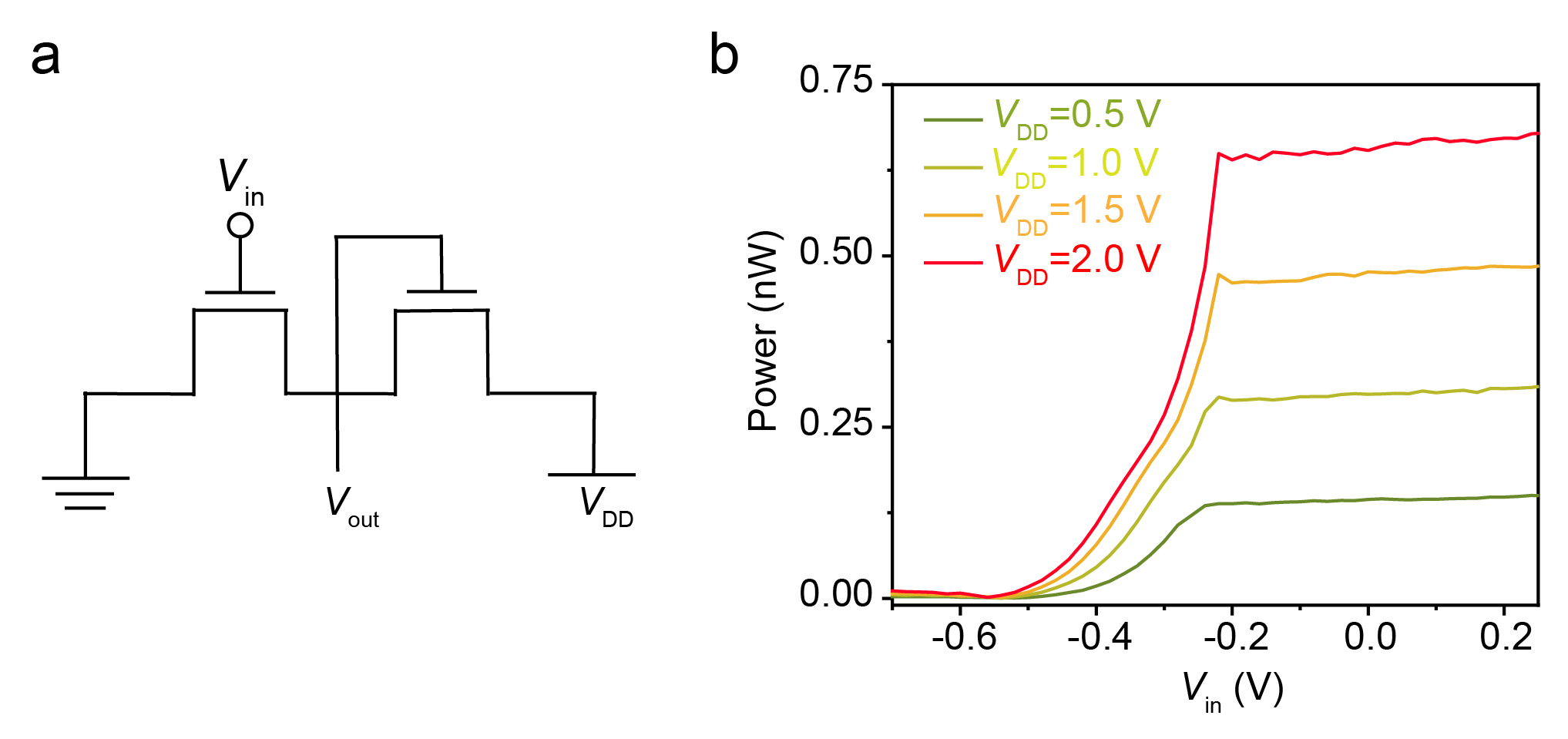


**Supplementary Fig. 26** | **Electrical performance of the inverter based on two top-gated Bi_2_SiO_5_/MoS_2_ FETs. a** Circuit diagram of the inverter. **b** Dynamic power consumption of the inverter.
